# Addressing Facts and Gaps in the Phenolics Chemistry of Winery By-Products

**DOI:** 10.3390/molecules22020286

**Published:** 2017-02-14

**Authors:** Nelson F. L. Machado, Raúl Domínguez-Perles

**Affiliations:** 1 Centre for the Research and Technology of Agro-Environmental and Biological Sciences, University of Trás-os-Montes e Alto Douro (CITAB-UTAD), Quinta de Prados, 5000-801 Vila Real, Portugal; nmachado@utad.pt; 2 Research Group on Quality, Safety and Bioactivity of Plant Foods, Department of Food Science and Technology, CEBAS (CSIC), Campus University, Edif. 25, Espinardo, 30100 Murcia, Spain

**Keywords:** grape, *Vitis vinifera* L., phenolic compounds, chemical structure, biological activity

## Abstract

Grape and wine phenolics display a noticeable structural diversity, encompassing distinct compounds ranging from simple molecules to oligomers, as well as polymers usually designated as tannins. Since these compounds contribute critically to the organoleptic properties of wines, their analysis and quantification are of primordial importance for winery industry operators. Besides, the occurrence of these compounds has been also extensively described in winery residues, which have been pointed as a valuable source of bioactive phytochemicals presenting potential for the development of new added value products that could fit the current market demands. Therefore, the cumulative knowledge generated during the last decades has allowed the identification of the most promising compounds displaying interesting biological functions, as well as the chemical features responsible for the observed bioactivities. In this regard, the present review explores the scope of the existing knowledge, concerning the compounds found in these winery by-products, as well as the chemical features presumably responsible for the biological functions already identified. Moreover, the present work will hopefully pave the way for further actions to develop new powerful applications to these materials, thus, contributing to more sustainable valorization procedures and the development of newly obtained compounds with enhanced biological properties.

## 1. Introduction

**Figure 1:**
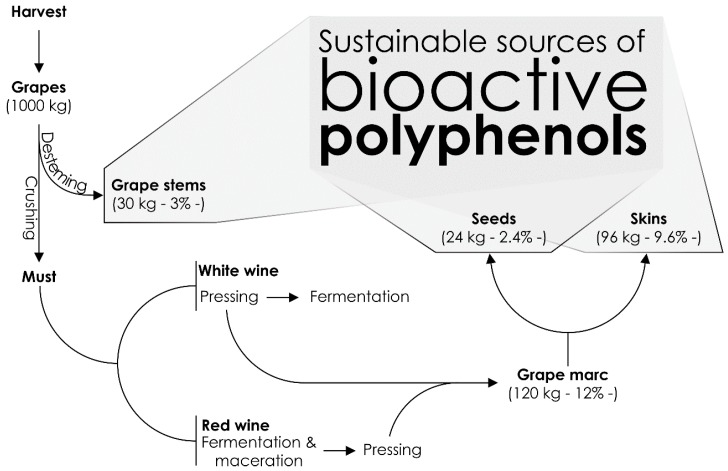
Flow diagram of vinification residues.

The organic fractions of agricultural residues represent a promising renewable resource for obtaining valuable compounds, which can be recovered through a myriad of processing alternatives. The winery industry, one of the most relevant socio-economic activities within the agro-food sector, is responsible for the exploitation of the large majority of grape (*Vitis vinifera* L.) production. This sector represents a worldwide extended agro-economical activity, which entails the production of noticeable quantities of by-products, with enough proven potential as to be surveyed and valorized as a sustainable source of bioactive compounds [1]. Hence, recent agro-economic estatistics have pointed out a world production of grapes amounting to 73.67 million metric tons in 2014, among which, 35.80 million metric tons (46.6% of the total) are directed to the winery industry [2], whereas up to 30.0% of the material used by this industry results in a diversity of by-products [3] (Figure 1). The generation of this biomass, consisting of grape pomace (seeds, skin, and peduncle), or isolated skins, as well as stems and lees, represents a current drawback of the utilization of grapes and wine production, in many countries, whilst the lack of proper pragmatic procedures for their valorization is considered a global issue, which merits to be addressed towards added value solutions for these materials. Therefore, achieving this objective will contribute to improved economic and environmental sustainabilities [4].

Consequently, public attention has been directed during the last decades to the limited capacity of local eco-environments to absorb the impact of food wastage. This growing awareness has been reflected in the resolution adopted on the subject by the European Parliament on 19 January 2012 (2011/2175(INI)), regarding the valorization strategies that should be implemented to avoid excessive wastage, making the food chain in the EU into more efficient. Indeed, this regulation has encouraged the fine-tuning of the production processes towards enhanced efficiency, alongside the development of new alternative uses for unavoidable wastes, which would contribute to reduce the dependency on raw materials.

Concerning the process of winemaking itself, the transfer of phytochemicals, such as phenolics, from grapes to must occurs mainly from grape skins, during the maceration step [5]. Thus, once in must and wines, these compounds are partially responsible for the sensory and biological attributions of wines [6]. Within this scope, regarding grape marc, the most abundant residue derived from the vinification process (accounting almost 20% of winery by-products), up to 65% of the phenolic compounds remains in grape marc after must extraction [7,8]. Nevertheless, phenolics are not the only bioactive compounds present in must, wines, and winery by-products. Hence, besides these compounds, the presence of additional non-phenolic, bioactive compounds, has been described till the present date, including terpenes, pyrazines, and norisoprenoids, as well as bioactive nutrients such as tocopherols and tocotrienols [6,9,10,11,12].

As stated recently by several autors a myriad of factors influence the polyphenolic content of grapes’ residues. In this sense, white and red grapes present qualitative (given the absence of athocyanins in white varieties) and quantitative (lower amount of phenolic compounds have been described in white varieties) polyphenolic composition. This fact should be taken into account when designing the extraction, purification, and valorization procedures for these molecules, as well as regarding the plant material that should be considered [3].

The functional composition of winery residues is attracting increased interest because, over the last three decades, almost 60% of the new drugs developed for medicinal purposes have been derived from natural products through structural modifications of compounds recovered from diverse natural matrices. These approaches, along with the direct synthesis of new compounds and structural analoges of nature bioactives have allowed researchers to shed some light on the structure-activity relationships (SARs) of compounds available in Nature [13]. Nevertheless, despite the number of natural products explored concerning functional properties, taking adavantage of their wide structural diversity, [14] the usage of these compounds for drug discovery purposes has decreased. This fact can be assigned, for instance, to the difficulties involved in setting up controlled cultures of wild plant species of interest, which are required to guarantee the consistency of the biological activity of their biofunctional compounds. Besides this constraint, the high extraction cost, the low yield of purified specific compounds with valuable properties, and the structural complexity of several compounds, which are often difficult to modify in lead optimization stages (or even to reproduce through directed synthesis), represent important issues to be considered in the process of drugs discovery or development based on natural matrices [15,16,17,18].

In contrast to the aforementioned situation, in the last recent years, the search for bioactive compounds based on the survey of natural products has undergone a resurgence driven by new technological breakthroughs, which have allowed a more efficient exploitation of natural compounds based on their chemical features and biological activities. Actually, the recent progress in molecular biology concerning technical and technological issues are breaking frontiers and expanding possibilities in the search for new bioactive molecules [18].

Furthermore, in what respects to the valorization of solid residues from the winery industry, not only the richness in bioactive compounds should be considered in order to design realistic valorization strategies, but also the presence of constituents with a negative contribution (mainly represented by tannins and fungal toxins) [19,20].

The environmental constraint represented by food wastage closely related to the agro-food chain has raised the public awareness in recent years, which has been mirrored in the resolution adopted on the subject by the European Parliament (2011/2175 (INI)). In this concern, recovering and using agricultural and food processing by-products are pragmatic procedures that can lower environmental harms associated to waste disposal and, simultaneously, optimize the use of the increasingly limited resources. Hence, earlier studies have estimated the extent of winery waste generation and the potential of by-product recovery as sources of food additives and/or nutraceuticals due to their richness on high-added value compounds [1]. Therefore, in the present review we will critically outline the challenges involved in finding new bioactive compounds in the distinct by-products from winery, as well as the rational drawbacks underlying their exploitation by either direct isolation or chemical modification. In this framework, the strategies to overcome the currently identified limitations to the exploitation of pollutant agro-food wastes as sources of bioactive compounds will be also reviewed, with the purpose of identifying cutting-edge applications or uses for these residues.

## 2. Chemistry of Winery By-Products

Chemistry of grapes and wines has been extensively studied in the last forty years, providing accurate information on the phenolic compound compositions shared between wine and winery by-products. Hence, the main differences identified seem to be attributable, besides the physiological features of the diverse grape plant tissues, to the chemical reactions occurring in must and wine during fermentation, as well as those arising in the plant materials during disposal until final processing. In this regard, as previously reviewed thoroughly by Teixeira et al., white and red varieties of grape produce solid residues displaying differentiated phenolic compound profiles [3], which will be detailed in the appropriate sections within this review, concerning specific sub-groups of phenolics.

Phenolic compounds form part of a group of secondary metabolites involved in plants’ responses against biotic and abiotic stresses [21], and are derivatives of the pentose phosphate, shikimate, and phenylpropanoid pathways in plants [22]. As a common distinctive feature, these compounds contain an aromatic ring with at least one hydroxyl group. This basic structure is complemented by the presence of additional moieties such as glycosides, esters, and methyl esters, among others, giving rise to other functional compounds with different properties concerning bioavailability and biological activity [23]. For this reason, the diversity of phenolic compounds present in winery industry wastage needs to be described in detail, concerning their relative concentration, chemical characteristics, and biological functions, as well as their SARs. However, this information does not constitute a final goal in itself, but an asset contributing to foreseen the actual scope of the valorization procedures envisaged.

General information already available concerning this issue allows stressing that the contents in individual phenolics, ascribed to the diverse classes, depend greatly on the winery residue considered, as well as on the extraction methodology, the winemaking process, the vintage, the maturity of fruits, the climate, and the grape variety under consideration (indeed the genetic factor is specially relevant when comparing white and red varieties) [24]. Moreover, since total extractable phenolics in grape represents up to 10% of pulp, 70% of seeds, and 35% of skin [25], the design and development of new uses of winery residues based on the isolation of phenolic compounds, has been envisaged. In addition to these residues, grape stems have claimed the attention of the scientific community in the last years, that has described phenolic concentrations ranging from 26.9 to 36.0 mg GAE·g^−1^ dw, with a valuable profile of compounds including hydroxycinnamic acids, flavonoids, and stilbenes [3].

Given the relevance of this issue, although during the last four decades, the valorization of agro-food by-products has been promoted though distinct official statements, been studied by scientists, and conducted by producers through the evaluation of the phenolic profile and radical scavenging properties of plant materials [3]. Nowadays, additional evaluations are still necessary, by applying improved analytical instrumentation available, which would allow gaining further insights in this issue, addressing the major benefits expected from these still underexploited plant materials.

Thus, phenolic compounds are the major class of bioactive phytochemicals in winery residues, being described in grape pomace, skins, stems, and lees, and belonging to three main classes: (i) phenolic acids (benzoic and cinnamic acids); (ii) simple flavonoids (flavanols, flavonols, and anthocyanins); and (iii) tannins and proanthocyanidins [26], whilst the evaluation of the compounds’ distribution in the separate residues has allowed to describe flavonoids as the predominant class in skins, seeds, and stems, and non-flavonoid phenolics the most important in the berry pulp [27], being consistent this relative occurrence of the diverse classes of phenolic compounds in white and red varieties. Hereafter, the qualitative and quantitative information available in the literature, regarding the phenolic composition of solid winery residues, is accurately described.

### 2.1. Phenolic Acids

Compounds belonging to this class are featured by a single carboxylic acid group, being subclassified into hydroxycinnamic and hydroxybenzoic acids. Within these subclasses, the number and position of the hydroxyl groups on the aromatic ring create the variety of structures and compounds described so far, which are featured by clearly differentiated bioavailability and biological activity [28]. In this connection, all hydroxybenzoic acids have a common C_6_–C_1_ structure and, concerning winery by-products, include the presence of gallic, hydroxybenzoic, protocatechuic, syringic, and vanillic acids [24,27,29,30,31,32,33,34,35,36,37,38,39], whilst hydroxycinnamic acids are aromatic compounds displaying a C_6_–C_3_ structure and accounting with caffeic, caftaric, chlorogenic, *p*-coumaric, coutaric, fertaric, and ferulic acids [27,29,31,36,37,38,39,40,41,42,43,44] (Table 1).

Hydroxybenzoic acids are unequally distributed in the different winery residues, with skins representing the by-product presenting a wider profile. Thus, whilst the phenolic compounds found in grape skins have been reported in the following decreasing concentration order: gallic acid > protocatechuic acid > vanillic acid > syringic acic > gentisic acid > hydroxybenzoic acid, till the present date, in seeds, only the presence of gallic, protocatechuic, and vanillic acids has been observed (Table 1). In respect to the quantitative relevance of these compounds, the concentration of the hydroxybenzoic acids in grape seeds is, in general, higher than in skins. In addition, in grape pomace, a winery residue encompassing grape seeds, skins, and pulp, the minority hydroxybenzoic acids are not found in the subresidues constituting grape pomace, if these are considered separately. This indicates a possible leaching of compounds from solid residues into the must during crushing and pressing. An additional factor contributing to the phenolic profile of grape pomace is the relative contribution of each material to the final phenolic composition. Grape stems have been noted as the solid residue derived from the winemaking process displaying the lowest number of hydroxybenzoic acids (gallic and protocatechuic acids), which are present in intermediate concentrations, respecting skins and seeds. In all cases, gallic acid is the most abundant hydroxybenzoic acid (Table 1), which relevance is due to its role as a precursor of hyrolyzable tannins [45]. The wide variation observed, regarding the concentrations of the major hydroxybenzoic acids described in these plant materials (gallic and protocatechuic acids), suggests that these compounds could undergo the effect of influencing factors (genetic background—including the type of grape variety—distinct agro-climatic conditions, and post-harvest processing), though analytical constraints should be also considered.

The additional group of phenolic acids described in winery residues, hydroxycinnamic acids, is generally present in lower concentrations than the hydroxybenzoic ones. The diversity of individual compounds within this phenolic class seems to be influenced by the grape tissue considered, in a lower extent than the hydroxybenzoic acid profile. Thus, grape skins are featured by containing caffeic, caftaric, chlorogenic, coumaric, fertaric, and ferulic acids, whilst chlorogenic acid is the major one with concentrations ranging from 0.29 to 231.10 µg·g^−1^ dw [43]. A similar profile has been found in grape seeds (with the exception of ferulic acid), even though the concentrations of phenolic compounds in this sub-residue are almost half of those observed in grape skins [38,43]. In opposition to the trend observed for hydroxybenzoic acids, grape pomace presented higher levels of almost all compounds within this class, indicating a lower influence of the vinification activities, respecting other phenolic classes. Additionally, the occurrence of these compounds in grape pulp [27], also allows considering this residue as a valuable source of these bioactive compounds. The detailed analysis of the information available in the literature shows that hydroxycinnamic acids in grape stems include caftaric acid (0.20–277.20 µg·g^−1^ dw), coutaric acid (6.10–63.40 µg·g^−1^ dw), fertaric acid (4.80–5.90 µg·g^−1^ dw), and ferulic acid (6.70–27.80 µg·g^−1^ dw), which has been described in similar levels to those observed for grape pomace (Table 1). Again, caftaric acid appears as the preponderant hydroxycinnamic acid in this residue [27,31,36,38].

With respect to the distinct phenolic profiles in white and red varieties, regarding phenolic acids, both types (hydroxybenzoic and hydroxycinnamic acids) count with representative compounds in red and white varities. Indeed, according to data available in the literature (Table 1), minor differences can be found regarding the phenolic acids, with protocatechuic and syringic acids being only described in red varieties to date [31,33,34,39], whilst the presence of fertaric and coutaric acids is restricted to solid residues from white varieties [36,38]. In this regard, it is important to stress that, even though almost all phenolic acids described in by-products are present in both types of grape plants, the concentrations described so far evidenced higher amounts of these compounds in residues obtained from red grapes.

### 2.2. Flavonoids

Flavonoids refer to a group of compounds constituted by low molecular weight molecules, structurally composed by 15 carbon atoms [46]. In solid winery industry residues, flavonoids are integrated by flavanols or flavan-3-ols, proanthocyanidins, flavonols, and anthocyanins (Table 2 and Table 3), which concentration in solid organic residues increases as a result of the augmented synthesis occurring during ripening [47]. These subclasses of flavonoids differ in the extent of the oxidation of the central pyran ring, as well as in the degree of polymerization [48]. Hence, flavonoids have been described in the aglycone or glycosidic conjugative forms, being characterized by different reactivity, as well as polarity, and thus solubility in different solvents [49]. These chemical features are closely related to the efficiency of the extraction processes, therefore, with the potential utilization of these compounds in the development of new, added value, functional products.

#### 2.2.1. Flavanols

Flavanols, also called flavan-3-ols, present a hydroxyl group at the C_3_ position and a carbonyl group at the position C_4_. Compounds belonging to this type of flavonoid act as attractants to pollinators and dyers in plants, also contributing to the organoleptic properties of plant foods. Additionally, flavanols constitute the most abundant compounds responsible for the astringency, bitterness, and structure of wines, being also present in winery residues, besides the musts [50]. They are classified into two distinct sub-categories: hydrolysable and condensed tannins [48]. Hydrolysable tannins are complex (poly)phenolic compounds that can be degraded into smaller units, mainly sugars and phenolic acids [51], whilst the chemical structure of condensed tannins (also named proanthocyanidins) varies depending on their constitutive flavanols, the degree of polymerization, and the linkage position [52].

Flavanol monomers react with each other, or monomer chains, to form proanthocyanidins, which are oligomers and polymers of flavanol units featured by complex chemical structures of low molecular weight, consisting of fifteen carbon atoms arranged in a C_6_–C_3_–C_6_ configuration, providing more than 6500 molecules [28,53]. These compounds are synthetized by the shikimate pathway, which is directly related to the lignin and sinapate ester biosynthesis, accounting of a number of branch pathways that lead to different subclasses including flavonols, proanthocyanidins, and anthocyanins [54].

The flavanol monomers described so far in winery residues are catechin and epicatechin, both of them present in grape seeds, skins, pomace, and stems. The analysis of the relative abundance of these two flavonols has allowed the identification of catechin as the most abundant in red and white varieties, with average concentrations almost 4-fold higher than epicatechin [24,29,30,31,32,34,36,38,41,43,55,56,57] (Table 2). This fact is supported not only by works developed for analytical purposes, but also with regard to evaluations of the impact of high voltage electrical discharges on the efficiency of the extraction of grape marc’s phenolics, which have provided values of catechin and epicatechin concentration in grape marc of 371–549 and 179–311 µg·g^−1^ dw, respectively [58]. These data suggest that, although there is a consensus on the ratio of both flavanols, the extraction conditions could affect the final efficiency of the process concerning the relative abundance of these compounds. This trend displays an opposite relationship between the relative amount of catechin and epicatechin [5,7,15,28,59], whilst no close associations with specific subresidues or the type of grape (white or red) are supported by data available in the literature (Table 2).

Data informing on the occurrence of hydrolysable tannins in winery by-products evidenced the presence of epicatechin gallate, epigallocatechin, epigallocatechin gallate, and gallocatechin gallate, with different frequence in the distinct residues (Table 2). Thus, whilst epicatechin gallate, epigallocatechin, and epigallocatechin gallate have been found in grape seeds, in skins the presence of epigallocatechin, epigallocatechin gallate, and gallocatechin gallate was detected. On the other hand, in grape stems and pomace only the presence of epicatechin gallate was described. Data on quantitative concentrations suggest that, generally, the highest concentration of all hydrolysable tannins is found in grape seeds, being epicatechin gallate the most abundant compound with values of up to 58.8 mg·g^−1^ dw [44]. Although this compound has been also found in grape pomace and stems, in these materials it has been detected at lower concentrations (micromolar level) (Table 2). Additional compounds within this class have been described in grape pomace and stems in decreasing concentrations, as follows: epigallocatechin gallate (up to 500.00 µg·g^−1^ dw) > gallocatechin gallate (up to 156.00 µg·g^−1^ dw) > epigallocatechin (up to 129.00 µg·g^−1^ dw). In skins, this trend is complemented with the occurrence of gallocatechin gallate (up to 2.04 µg·g^−1^ dw) and epigallocatechin gallate and epigallocatechin (0.45 µg·g^−1^ dw, on average) (Table 2).

Despite the wide diversity of flavanols identified in winery residues, nowadays, there is sturdy evidence on the leading role of proanthocyanidins as parent compounds of the most abundant phenolics in these materials. Within proanthocyanidin compounds, dimeric, trimeric, and tetrameric compounds are often referred as B-, C-, and D-series, respectively. Thus, the evaluation of these materials has allowed the description of the presence of nine different dimers (procyanidins B1, B2, B3, B4, B5, and B7, and the procyanidin derivatives B1-, B2-, and B4-gallate), four trimers (C1 and C2, as well as C1- and C2-gallate trimer derivatives), and two tetramers (D1 and D2). Dimers and trimers are composed of catechins and epicatechins [25].

Proanthocyanidins or condensed tannins account for several subclasses of compounds distinguished on the basis of the hydroxylation patterns of their constitutive units and the linkages between them. These compounds are phenolics containing up to 20 flavanol units, whilst the most common constitutive units are (epi)catechins and (epi)gallocatechins, indicating procyanidin and prodelphinidin structures, respectively [60]. Procyanidins are members of the proanthocyanidins class, constituted by oligomers or polymers of flavanols that exclusively consist of catechin and/or epicatechin units [61], and by esters of gallic and ellagic acids, giving rise to gallo- and ellagitannins, respectively [62]. Because of their chemical properties tannins bind with proteins, basic compounds such as alkaloids, and heavy metallic ions in solution, which make them insoluble [63]. These chemical reactions compromise their utilization in the development of functional products, decreasing their biological value, as well as the bioavailability of diverse biologically interesting compounds. Additional valuable flavanols present in grape seeds are the (+)-gallocatechin, (−)-epigallocatechin, and (−)-epigallocatechin gallate based procyanidins [25]. This flavonoid subgroup is the most abundant subtype of proanthocyanidins occurring in grape seeds, being chemically constituted by the flavanol units (−)-epicatechin and/or (+)-catechin linked by C_4_–C_8_ or C_4_–C_6_ bonds (type-B structures), or doubly linked by an additional C_2_–C_7_ ether (acetal) bond (type-A structures). These compounds may include gallic acid esterified at the C_3_ position of the flavanol residues. In seeds, these compounds are mainly found as dimer, trimer, and tetramers, the highest level of polymerization being described as heptamers [64,65].

With respect to procyanidins, the B1 type has been found at higher concentrations (10.60–1958.00 µg·g^−1^ dw) than the B2 (3.00–910.00 µg·g^−1^ dw) subtype in all sub-residues produced during the vinification process (Table 2) as well as in both white and red varieties, whilst large differences have been found, depending on the analytical technique used for their detection. In this respect, the highest concentrations have been reported when resorting to HPLC-DAD instrumentation, in comparison to HPLC-DAD-ESi-MSn (Table 2). Besides these two major procyanidin dimers, to the present date, the occurrence of four additional dimers has been also described. Procyanidin dimer B3 presented the highest level in grape stems (20.00–993.00 µg·g^−1^ dw), with lower concentrations (0.60–350.00 µg·g^−1^ dw) seen in grape skins, seeds, and pomaces. The presence of procyanidin B4 has been also described in all four matrices, with grape pomace representing the most relevant source (17.00–515.20 µg·g^−1^ dw), followed by grape seeds and skins (2.00–310.00 µg·g^−1^ dw), which reach 2- and 4-fold higher concentrations, with respect to those described in grape stems (Table 2). Finally, procyanidin B5 and B7 have been also described in grape seeds and pomace, respectively, in concentrations of up to 356.70 and 90.00 µg·g^−1^ dw, respectively (Table 2). The description of gallate derivatives of the procyanidin dimers has been based on the finding of three different compounds (procyanidins B1-, B2-, and B4-gallate), from which the B1-derivative is only absent from grape pomace, whilst the B2 derivative has been found in all residues, in values ranging from 23.00 to 1372.90 µg·g^−1^ dw. The description of procyanidin B4 gallate has been only referred concerning grape seeds, being present in concentration ranges between 9.20 and 20.80 µg·g^−1^ dw (Table 2).

Procyanidins trimers and tetramers have been also described in all winery residues as referred in Table 2. In this regard, trimers C1 and C2 have been found at the highest concentration in grape seeds (up to 600.00 and 476.00 up to µg·g^−1^ dw, respectively) followed by grape pomace (up to 201.10 and 449.70 µg·g^−1^ dw, respectively). In addition, the presence of the procyanidin C1-gallate derivative has been described in grape seeds, with a concentration fluctuating from 15.10 to 31.90 µg·g^−1^ dw. Interestingly, procyanidin tetramers D1 and D2 were also described in these materials, even though their relative abundance was closely dependent on the considered sub-materias, the highest concentrations corresponding to grape pomace, followed by grape seeds and stems, which remained in similar lower levels (Table 2).

According to this information, it is widely accepted that hydrolysable tannins represent the most abundant subtype of phenolics occurring in grape seeds, being chemically constituted by the flavanol units (−)-epicatechin and/or (+)-catechin linked by C_4_–C_8_ or C_4_–C_6_ bonds (type-B structures), or doubly linked by an additional C_2_–C_7_ ether (acetal) bond (type-A structures) [64]. Indeed, the relative value of the separate winery residues as sources of catechin and epicatechin has been supported by a higher content in seeds, in comparison with skins, with catechin, epicatechin, and epicatechin gallate, being the most abundant compounds in both matrices [31]. However, once again, the combined occurrence of diverse factors is responsible for the final yield and chemical composition of polyphenolic extracts in these grape matrices. In this regard, Slier et al. have reported a significant effect of the ripening stage in the concentration of phenolic compounds in grape plant materials, causing a reduction of the concentration, from the initial formation of the fruit, to full ripe grapes used in the wine production.

The evaluation of the data reported in the literature, concerning the diverse profiles of flavonoids in residues obtained when processing red or white varieties, shows again a lack of critical differences between both types of grape varieties, since the qualitative determinations performed so far reported almost the same panel of compounds. However, when considering the quantitative concentration of these compounds, it can be pointed that red varieties account with significantly higher concentrations of bioactive phenolics [39]. In this regard, caution has to be made regarding this observation, since, as demonstrated by Pantelic and col., these differences seem to be enclosed to the specific material under evaluation. Hence, when Pantelic et al. evaluated the polyphenolic content of winery residues from red and white varieties, resorting to a PCA approach developed with data on phytochemical composition, this did not allow discriminating between materials, given the similarities between some of them [39].

#### 2.2.2. Flavonols

This constitutes a subclass of flavonoids recurrently identified in winery by-products, which are chemically characterized by the presence of a double bond between carbons C_2_ and C_3_, and a hydroxyl group in C_3_ [70]. These phenolic compounds are found in the aglycone form or bound to glucosides, glucuronides, galactosides, and diglycosides (glucosylarabinoside, glucosylgalactoside, glucosylxyloside, and glucosylrhamnoside) [68]. They are differentially distributed in the diverse plant matrices, concerning winery residues, in which respects to their occurrence and relative abundance (Table 3).

In winery by-products, flavonols are mainly represented by isorhamnetin, kaempferol, quercetin, and syringenin, as well as their glycoside derivatives (Table 3). The largest diversity of flavonols has been described in grape pomace (*n* = 25), followed by grape skins (*n* = 14) and stems (*n* = 11), whilst grape seeds display a lower assortment (*n* = 6). In respect to their chemical form, the higher quantities correspond to glycoside molecules, which present varied concentrations depending on the material considered, whilst aglycones (kaempferol, myrcetin, and syrengin) are present in much lower concentrations, or even absent. Thus, only aglycone quercetin has been described in relative amounts presenting similar levels to glycoside derivatives, although this situation is restricted to grape pomace (Table 3).

The detailed examination of the quantitative data available denotes quercetin derivatives as the most abundant compounds in all materials (Table 3). Thus, regarding the relative abundance of quercetin derivatives in grape pomace, it has been found that quercetin-3-*O*-glucoside and quercetin-3-*O*-glucuronide represent the most abundant compounds, with concentrations ranging from 26.00 to 704.00 and from 31.99 to 522.50 µg·g^−1^ dw, respectively. Besides, these values are much higher in comparison to those observed in skins, seeds, and stems, in which values up to 200.00 µg·g^−1^ dw have been described (Table 3). Another abundant quercetin derivative (quercetin-3-*O*-rutinoside) has been described in similar concentrations, in grape skins and pomace, where levels of up to 570.40 µg·g^−1^ dw have been observed, while its concentration in grape seeds and stems ranges between 0.40 and 90.50 µg·g^−1^ dw (Table 3).

Isorhamnetin derivatives constitute the second major froup of flavonols in winery residues. This group is represented by four derivatives, namely the aglycone isorhamnetin, isorhamnetin-3-*O*-glucoside, isorhamnetin-3-*O*-(6-*O*-feruloyl)-glucoside, and isorhamnetin-3-*O*-glucuronide [31,35,36,42,57,66]. In grape pomace, in which all derivatives of isorhamnetin identified so far are present, the most abundant is isorhamnetin-3-*O*-glucoside, with concentrations ranging from 3.20 to 63.80 µg·g^−1^ dw (on average 3-fold and 6-fold higher in comparison with isorhamentin and isorhamnetin-3-*O*-glucuronide, respectively). In grape skins, isorhamnetin derivatives are represented by the corresponding glucoside (1.00–23.10 µg·g^−1^ dw) and glucuronide derivatives (3.50–9.40 µg·g^−1^ dw), whilst in grape stems the presence of isorhamnetin-3-*O*-(6-*O*-feruloyl)-glucoside (6.90–9.10 µg·g^−1^ dw) has been also described (Table 3).

Kaempferol derivatives constitute a valuable type of bioactive phenolics widely reported in grape residues as well. Regarding these compounds, the aglycone form is present in grape skins, stems, and pomace, in concentrations up to 13.60, 15.50, and 34.20 µg·g^−1^ dw, respectively. On the other hand, the glucoside and galactoside derivatives (ranging from 1.50 to 100.00 and from 0.10 to 47.40 µg·g^−1^ dw, respectively) are the major representatives in all materials, followed by the glururonide derivative (1.00–13.10 µg·g^−1^ dw) (Table 3). Contrary to other subtypes of flavonols, the highest concentration of these compounds is not always found in grape pomace, but rather in seeds and skins (kaempferol-3-*O*-glucoside) and stems (kaempferol-3-*O*-glucuronide and kaempferol-3-*O*-rutinoside) (Table 3).

Other minority flavonols present in grape by-products are represented by laricitrin (grape pomace), syringetin (grape pomace), and myricetin (grape skin and pomace) derivatives, present in concentrations ranging from 0.10 to 6.40, from 0.50 to 13.80, and from 0.40 to 12.00 µg·g^−1^ dw (Table 3).

#### 2.2.3. Anthocyanidins

A very relevant group of flavonoids present in grapes and by-products, and restricted to plant material from red varieties, is constituted by the anthocyanidins, formed by glycosides of polyhydroxy and polymethoxy derivatives of 2-polyphenylbenzopyrylium or flavylium salts [53]. These compounds are highly hydrosoluble and featured by characteristic red color properties in addition to the biological activities recognized to phenolic compounds. Indeed, anthocyanins are responsible for most of the red, blue, and purple colors in several plant tissues according to their chemical structure (degree of hydroxylation, methylation, and glycosilation), although they exist mainly in the stable red coloured form (the flavylium cation form) at low pH values [51]. Generally, anthocyanidins are found in the form of glycosides (anthocyanins), whilst aglycones are rarely found [28].

Compounds withing the anthocyanin subclass of flavonoids (cyanidin, delphinidin, peonidin, petunidin, and malvidin, among others) include an aromatic ring (A) bonded to an heterocyclic ring (C) that contains oxygen, which is also bonded by a carbon–carbon bond to a third aromatic ring (B). This aromatic ring B forms conjugates with sugars and organic acids to generate a multitude of anthocyanidins of diverse colors. These compounds are relatively unstable and easily oxidized, which modifies their chemical properties, reducing their biological interest [3]. They are sensitive to many factors, apart from pH, that may affect their stability and color, such as temperature and UV radiation [71]. These chemical features have prompted the ascription of promising technological and biological applications to these compounds.

When comparing the separate residues obtained as a side consequence of the vinification process, the most valuable one is grape skin, in which all types of anthocyanidins (aglicones and glycosylated forms) described in grapes have been found. Besides, grape pomace contains only anthocyanin glycosylated, acylated, and coumaroyl derivatives. Interestingly, grape stems have been recently described as an interesting source of anthocyanins, even though only three anthocyanidins (malvidin-3-*O*-glucoside, malvidin-3-*O*-rutinoside, and malvidin-3-*O*-(-6-*O*-caffeoyl)-glucoside have been found to the present date [42,72].

The most abundant anthocyanins described in grape skins are found in the following decreasing order: malvidin-3-*O*-glucoside > malvidin-3-*O*-acetylglucoside > petunidin-3-*O*-glucoside > peonidin-3-*O*-glucoside > delphinidin-3-*O*-glucoside > cyanidin-3-*O*-glucoside > malvidin-3-*O*-*p*-coumaroilglucoside > petunidin-3-*O*-acetylglucoside > delphinidin-3-*O*-acetylglucoside > peonidin-3-*O*-acetylglucoside > cyanidin-3-*O*-acetylglucoside > cyanidin-3-*O*-*p*-coumaroylglucoside > peonidin-3-*O*-*p*-coumaroylglucoside > petunidin-3-*O*-*p*-coumaroylglucoside (Table 1) [35] (Table 4).

The comparison of the quantitative occurrence of anthocyanins in grape residues denotes that even though malvidin has been identified as the major anthocyanidin in grapes, a critical revision of the recent bibliography allows one to refer the glycosylated derivatives of petunidin-3-*O*-glucoside (507.60–684.80 µg·g^−1^ dw), peonidin-3-*O*-glucoside (45.80–220.40 µg·g^−1^ dw), delphinidin-3-*O*-glucoside (39,30–142,10 µg·g^−1^ dw), and malvidin-3-*O*-glucoside (51.70–124.80 µg·g^−1^ dw) as the most abundant [35]. With respect to acylated derivatives, those from petunidin are again the most abundant, displaying values of up to 300.70 µg·g^−1^ dw [35]. Additional acylated anthocyanins of peonidin, malvidin, and delphinidin (9.70–36.20, 12.20–29.80, and 2.20–15.50 µg·g^−1^ dw) have been found, in much lower concentrations, in skins. Finally, a similar pattern of the coumaroyl anthocyanidins has been described, and again, the petunidin derivative is the most abundant, with values ranging from 21.90 to 33.90 µg·g^−1^ dw, followed by cyanidin-3-*O*-coumaroyl-glucoside (2.10–8.90 µg·g^−1^ dw), peonidin-3-*O*-coumaroyl-glucoside (0.70–7.50 µg·g^−1^ dw), and malvidin-3-*O*-coumaroyl-glucoside (0.60–3.30 µg·g^−1^ dw) [35,44]. Interestingly, to date, no coumaroyl derivatives of delphinidin have been described in grape skins.

The comparison of the information available concerning the anthocyanin content of skins, with those describing the composition of grape pomace, evidenced a change in the profile of both materials. Such a situation can only be attributed to chemical reactions and interconversions between compounds occurring during the crushing process, and after the disruption of the grape structure, when anthocyanidins are susceptible to be involved in diverse chemical reactions, whilst the physical and chemicals conditions change severely. Thus, in grape pomace, the most abundant anthocyanin is malvidin-3-*O*-coumaroyl-glucoside that is present in concentrations of up to 238.90 µg·g^−1^ dw, followed by malvidin-3-*O*-acetyl-glucoside (up to 195.00 µg·g^−1^ dw), and malvidin-3-*O*-glucoside (142.20 µg·g^−1^ dw) (Table 4). As aforementioned, in this case, malvidin effectively appears as the most abundant colored phenolic, reinforcing the idea of the modifications in the anthocyanidins profile due to the vinification process (especially because grape pomace represents the most extensively studied residue). In addition to malvidin derivatives, in grape pomace, high levels of petunidin-3-*O*-coumaroyl-glucoside, delphinidin-3-*O*-coumaroyl-glucoside, peonidin-3-*O*-coumaroyl-glucoside, and malvidin-3-*O*-caffeoyl-glucoside have been also described [44].

#### 2.2.4. Stilbenoids

Stilbenes (also named 1,2-diarylethenes) are phenolic compounds with promising health effects due to their potential biological attributes, chemically constituted by two aromatic rings linked by an ethylene bridge and with three hydroxyl groups in the aromatic ring [51], which can be found in winery by-products (Table 5), that have attracted the interest of the ‘hunters’ of new bioactive compounds. Within this group, the presence of resveratrol (3,5,4′-trihydroxystilbene), a monomeric compound that constitutes the major stilbene in grapes [73], is of special relevance. Other compounds within this group, also found in the winery residues, have been considered as candidates to be evaluated on their biological power, namely *trans*-piceid (*trans*-resveratrol-3-*O*-glucoside), and ε-viniferin [74]. In this regard, the major concentrations of stilbenes have been attributed so far to grape stems, to which values of up to 6400.00 µg·g^−1^ dw—(*trans*-picied) or 1400.00 µg·g^−1^ dw (*trans*-resveratrol) have been observed. Moreover, in grape stems, it have been also found interesting concentrations of *trans*-picied and ε-viniferin, reaching up to 266.00 and 499.00 µg·g^−1^ dw, respectively. Finally, in grape pomace, minor concentrations of trans-resveratrol of up to 64.00 µg·g^−1^ dw have been described (Table 5).

When sorting out the available bibliographic references allowing one to understand the differences existing between red and white varieties, concerning the content in the different types of phenolic compounds, again, it can be elated that red varieties display significant higher concentrations [39,55], although some critical differences should be analyzed in each particular situation, given that some compounds such as ε-viniferin could be present in higher concentrations in white varieties [42]. Therefore, specific valorization procedures should be envisaged for red and white varieties, depending on the pursued goals.

Given the overall phenolic composition of winery residues, plant materials forming part of vinification wastes display an indubitable interest as a source of bioactive phytochemicals, while, to the present date, the phenolic compounds have been only partially evaluated for their biological potential. Hence, in order to shed some light on the valorization of these materials and compounds, it should be stated that the extraction conditions (supercritical fluids and modified co-solvents, among others) represent critical factors influencing the efficiency of the extraction processes and the occurrence of phenolic compounds in the obtained extracts. In this concern, new studies applying the recent knowledge gained towards more efficient extraction alternatives are required to identify the actual chemical diversity [76].

Furthermore, nowadays there is a consensus on the critical impact of the agro-climatic conditions on the phenolic profile and the relative amount of the major phenolics, in both grapes and winery wastes. However, this is not only referred to the soil features and climate conditions in the diverse geographical areas where the studies are conducted, but also to drawbacks enclosed to the current changing climate [59]. Indeed, this new situation is causing an increased awareness on quality monitoring as an essential stage of the valorization of wine industry by-products as sources of bioactive phytochemicals, since these changes entail a variation in the proportions of the polyphenolic compounds available in these matrices, conditioning the requirements of extraction and purification processes [77].

Nonetheless, although this fact could have a critical relevance for the appearance of bioactive compounds in the latter ripening stages, the significance of this information for the decision-making on the optimal stage for valorizing these residues is conditioned, in a higher extent, by the requirements of the wine production features. This fact is reinforced by the observed tendency of the content in oligomeric flavonols to decrease and the content in monomeric flavanols to increase during maturation [34]. In this sense, once those compounds with specially attractive bioactive and bioavailable features to be used in the development of new products are identified, the efforts should be focused on the identification of the grape varieties with higher interest at the ripe stage (as generally used in the winery process), besides the fine-tuning of the extraction processes, thus, allowing the profitable utilization of the compounds of interest.

## 3. Use of Chemometrics for the Traceability of Winery By-Products

Nowadays, the application of chemometrics for the traceability of distinct matrices, either by-products, foodstuffs, or even foods and beverages is gaining attention concerning the monitoring of different features. This kind of multivariate approach is generally applied in tandem with spectroscopic techniques, or resorting to specific parameters, which can be related with differences between samples, allowing the development of either discriminant/classification models, or methods for the evaluation of these matrices, resorting solely to the spectral acquisition, once proper models are calibrated and validated for each specific matrix [78,79].

In this kind of approach, a set of samples is assessed concerning specific contents or features, which are subsequently related to the diverse samples, thus allowing the exploitation of these differences, resorting to multivariate analysis that can be applied for distinct purposes [78,79,80,81]. In order to do so, the results previously collected for the samples can be explored without introducing a priori known membership, with unsupervised methods, such as Principal Component Analysis (PCA), which allows the observation of unforeseen similarities between samples, as well as the detection of outliers through the plots of the main principal components (PCs) extracted [82]. Furthermore, this approach can be also applied solely to reduce dozens or hundreds of variables to a few PCs [81].

Additionally, supervised methods can be also applied, such as Partial Least Squares-Regression (PLS-R), an iterative procedure where the response of interest (e.g., membership, specific content) is taken into account in the regressive procedure to extract the factors (which play the role of PCs in this methodology) [78]. Therefore, in this case, the variability extracted through this methodology, expressed in the factors’ scores, is directed to specific traits of interest. Generally, these approaches, reducing the variability of the samples into a strict set of factors, are known as Factorial Analysis (FA) techniques. Moreover, PLS can be also used for discrimination and classification purposes, resorting to PLS-Discriminant Analysis (PLS-DA), in which a set of samples, with previous known memberships, is used to define models that will subsequently allow the classification of ‘external’ samples for a calibrated parameter (grape variety, vintage, geographical origin, or period of storage, among others), which is of special relevance for traceability purposes, concerning fraud and adulteration [78,83]. Additionally, besides this ‘formal’ classification system, also hierarchical cluster analysis (HCA) can be applied, allowing to observe the similarities between distinct samples at different levels of familiarity [82,84]. Finally, more recent non-linear regression and classification models, such as Artificial Neural Networks (ANN) and Support Vector Machines (SVM) have been lately applied in winery, including the assessment of by-products [85,86].

In this issue, several works have been developed in the last years regarding the application of multivariate analyses for the traceability of winery by-products, the majority resorting to data from spectroscopic techniques [79,81,85]. Moreover, there are also works developed using specific parameters assessed as variables for prediction, such as the work of Bustamante et al., where several physico-chemical and biological parameters have been used as variables for the construction of models allowing the classification of composts obtained from winery and distillery wastes, concerning their maturity and quality [80]. This work allowed reducing the number of determinations needed to ascertain the maturity and quality of the composts, resorting to discriminat analysis, with a percentage of success around 95% [80]. A similar approach has been also used to assess the microbiological burden of these composts, besides the same physico-chemical parameters, in another work, which applied FA alongside HCA, showing that the composting system did not influence the final characteristics of the composts [84].

Furthermore, and closely related with the type of compounds reviewed in the present work, these chemometrical approaches can be also applied to the polyphenol profile, in order to classify samples. For instance, in the work of Figueiredo-González and colleagues the specific patterns of three grape varieties have been used as fingerprints for their ulterior identification based on specific contents, resorting to PCA and HCA [82]. In this work, it has been observed that some conjugates of delphinidin, malvidin, and peonidin represent useful anthocyanidins or anthocyanins for distinguishing selected varieties, while concerning flavonols, some conjugates of kaempferol, syringetin, myricetin, and quercetin could be used for discrimination purposes [82]. Since some specific contents allow the discrimination of samples, regarding a feature of interest, whereas it is well known that the signal observed in spectroscopic techniques reflects the composition of a sample, one approach that has been thoroughly explored relies on the use of such techniques, in tandem with multivariate analyses, mainly resorting to vibrational spectroscopy and NMR [79,81,85].

In which respects to vibrational techniques, Infrared Spectroscopy (IR) represents the one receiving more attention, due to its simplicity, mainly when applied with Fourier Transform Infrared Spectroscopy (FTIR) instruments, which allow the use of Attenuated Total Reflectance (ATR) and Diffuse Reflectance−FT (DRIFT) accessories for Mid and Near-IR (MIR and NIR), respectively. These analytical alternatives allow the registration of IR spectra for the raw materials, without any kind of sample preparation, or use of solvents, being thus especially suited for the winery industry [79,81,83]. Regarding the application of vibrational techniques to this specific industry, their potential has been widely proven by numerous applications in the last decades, accomplishing numerous purposes throughout all processes related to viticulture and vinification, from the management of soil practices to the analysis of bottled wine, including the assessment of grapevine leaves, grapes, and other tissues, as well as distinct aspects of the winemaking process, presenting different levels of success for the distinct applications. Although Raman spectroscopy has been less employed, mainly due to its added cost and complexity, the number of articles reporting the use of this technique have increased in the last few years, revealing its potential suitability for the winery industry demands [79,83].

Additionally, between the two distinct IR intervals used, assessed with resort to different instruments, NIR is the one that has gained more attention, due to its reliability and easiness of use, nowadays existing portable spectrophotometers for this specific interval, which are suitable for the winery industry, both for industrial and vineyard contexts [81,86]. Actually, NIR spectroscopy has been already successfully applied to winemaking residues, namely grape pomace, to estimate total phenolics content and total antioxidant capacity (*R*^2^ higher than 0.95), representing a non-destructive technique to assess the value of these residues [87]. The NIR hyperspectral imaging (900 and 1700 nm), resorting to portable cameras, has also been explored in the characterization of grape seeds, skins, and stems [79], besides being also used to discern grape varieties [86], resorting to ANN approaches in the latter case. Furthermore, NIR spectroscopy has been stressed as an analytical technique with enough potential for the quantification of total phenolics in grape skins throughout maturation [88], while NIR hyperspectral imaging represents nowadays a routine procedure to evaluate the maturity of grapes in industrial environment [83].

Concerning the application of these approaches to winery residues, the feasibility of FT-NIR spectroscopy to predict the extractable content of phenolic compounds directly in intact grape seeds has been recently reported by Torchio et al. in a work where the phenolic composition of 40 grape samples assessed by reference chemical methods, has been used for calibration. The results from this work have shown that a good predictive accuracy was only achieved when samples from two distinct years were simultaneously considered [89]. In this work, the models developed were sufficiently robust for quantitative purposes for total flavonoids, pro-anthocyanidins, low molecular weight flavanols, catechin, epicatechin, and procyanidin (Standard Error in Prediction—SEP < 15%), as well as for the assessment of the galloylation percentage, which shows the feasibility of this approach to assess winery residues, regarding valorization procedures [83,89].

In another work, undertaken with the same methodology, FT-NIR has been evaluated and compared with instrumental texture parameters associated with the content of total phenols and extractability predictors in intact Cabernet-Sauvignon seeds, from grapes harvested at six different advanced physiological stages throughout ripening. In this work, the best prediction of phenol content in the seeds, was found using FT-NIR spectroscopy in transmittance mode, corresponding to a SEP smaller than 8%, being much better than the SEP for phenol extractability [90]. Furthermore, NIR hyperspectral imaging has been also used to determine flavanols, and their extractability, in seeds of red and white grapes, using one cv. of each type. The PLS regression approach was applied, yielding *R*^2^ of 0.73 for total flavanol content and *R*^2^ of 0.85, respecting the correlation between analytical and predicted values, for flavanols extracted using a model wine solution, whereas higher *R*^2^ values (0.88) were observed when each one of the grape cultivars was analyzed separately [83].

Concerning the use of NMR for the assessment of these by-products, its application is not so widespread, since this technique requires obtaining an extract, if the sample is solid, which has to be redissolved in a deuterated solvent. Moreover, this technique is rather complex, comparing with IR, which compromises its suitability for the winery industry. Nonetheless, Fotakis et al. have published a thorough review in 2013, surveying metabolomic studies on grape derived products, such as berry, must, wine, vinegar, and grape marc distillates, resorting to NMR coupled to multivariate analysis. In this work, it was pointed that the NMR-based works concerning the *Vitis* species have framed metabolic patterns regarding the grape cultivar, terroir expression, vinification processes, organoleptic characteristics, and phytopathogenic reactions. Furthermore, a host of metabolic markers could be elucidated for the fermentation procedure on the grape substrate, which can be of special relevance for the assessment of by-products [85].

Nevertheless, in this review, the only work dealing with such residues was focused on the metabolite profiling of Greek grape marc spirits, resorting to the NMR technique, such liquor representing a common exploitation for these residues [85]. In this work it was shown the versatility of NMR to gather information from grape derived products, independently from their production procedure. A database has been created, based on the acquired spectroscopic data, which could constitute a reliable authentication tool for the determination of the geographical origin of grape marc distillates, besides allowing to discriminate markers that ascribe to genotypic diversity, production procedure and the vintage year [91].

## 4. Processing Alternatives: Challenges for Improving Phytochemical Profile

### 4.1. Storage

With the perspective of augmenting the value of such underexploited materials, given the seasonality of the winery activity and the disposal of the related by-products, their polyphenolic extracts often require a preservation period to be further used in valorization processes towards specific applications. In this sense and to avoid deleterious effects associated to stowage, the storage as dried extracts merits to be considered, since it consitutes a physical state featured by several advantages in comparison with solutions in extraction solvents. Such advantages include greater stability of active phytochemicals and lower storage costs, besides the versatility of these solids to be further processed towards the isolation and purification of compounds with proven value as biologically active molecules, or to be integrated in further processes at the required concentrations for the development of new functional products (foods or cosmetics). In this concern, one of the most widely used drying techniques by the food industry and others is spray-drying, a technique optimized by Pérez-Serradilla et al. This technique results in a procedure that allows one to obtain antioxidant wine lees’ extracts in the form of powder, presenting similar physicochemical characteristics to those of grape seed extracts [3,92].

Furthermore, concerning this issue, other authors have focused the research priorities on the combination of distinct procedures to improve the storability of these residues. Regarding this issue, Augustine et al. evaluated the simultaneous usage of low temperature, gamma radiation, and sodium benzoate (0.1%). This treatment allows the maintenance of the acidity required to preserve the stability of anthocyanins [3,93]. In the aforementioned work, from Augustine et al. the treatment with 0.1% SB and 2 kGy of radiation was found to be the most effective in maintaining anthocyanins stability, improving the storability of grape pomace conserved at low temperature conditions (4 ± 1 °C; 90.0%–95.0% relative humidity). The information reported by these authors suggests that this treatment would allow to preserve the value of grape pomace as a source of polyphenols, allowing 2-fold higher storage period in comparsion with untreated samples (16 vs. 8 days of storage), with a better recovery of anthocyanins [93].

### 4.2. Processing

Since the low concentrations of bioactive compounds often represent one of the major drawbacks, regarding the exploitation of bioactive molecules from winery residues, the enhancement of the phytochemical content of these matrices represents an interesting strategy, either aiming to the formation of new compounds, or to increase the yield of some previously existing phytochemicals. Besides, also the induced degradation of complex polysaccharides present in winery residues should been taken into consideration, not only making them available for animal nutrition, but also allowing a more efficient liberation of bioactive compounds of interest (polyphenols), which might be trapped, mainly by lignins [94,95]. Also the destruction of harmful compounds, such as toxins, or the reduction of the microbiological burden in these matrices, constitute approaches to account for, whilst these processes sometimes occur as a side effect of improvement treatments used when pursuing other goals. Moreover, the maintenance of the original richness of these matrices represents also an important asset, concerning the recovery of phytochemical (or other) compounds, besides the fine-tuning of the extraction processes. Therefore, it is important to protect the compounds to be extracted from the degradation associated with the manipulation of the target materials till the processing or extraction steps, keeping their recovery profitable, and thus sustainable [94].

#### 4.2.1. Fermentation Processes

In addition to compounds naturally occurring in grape materials, some of the molecules found in wine arise from the fermentation process, corresponding to metabolites resulting from the yeast or bacterial metabolization of compounds present in winery residues. These compounds contribute largely for the differentiation between distinct wines—either concerning negative or positive features. In this sense, the winery industry residues may also be subjected to fermentation processes, which often occur spontaneously, depending on the storage conditions, while this treatment, leading to an array of new compounds, can be directed resorting to specific strains of yeasts or bacteria [10,96,97]. On the other hand, the destruction of non-desirable micro-organisms—using radiation, temperature, or controlling agents—also represents an important component of these treatments, avoiding the spoilage of the residues, which are thus decomposed solely by the strains that have been chosen, and are meant to do so [93,96]. Moreover, it is worth mentioning that in some cases, such as lactic acid bacteria, the polyphenols present in these residues display a protective role as defensive agents for the bacteria, reducing the effectiveness of certain controlling agents, such as lysozyme [98].

Concerning the usage of these alternative treatments to improve the residues’ composition, processing these by-products through fermentation has shown remarkable potential to improve the yield of extraction, besides changing the phytochemical profile, which occurs mainly due the modification of the cell wall structure by microbial enzymes produced during fermentation, leading to the liberation of bound phenolic compounds, while the microbial metabolism of these compounds also leads to the formation of a large array of new metabolites [96,99]. Concerning these metabolites, they can be formed through different bioconversion pathways such as glycosylation, deglycosylation, ring cleavage, methylation, glucuronidation, and sulfate conjugation, whilst their occurrence depends on the strains and substrates involved. Moreover, even though the metabolic pathways related to the formation and bioactivities of these metabolites are highly underexplored, besides their quantification, this strategy displays enough potential as to be used in the production of extracts with a high-added value, from these organic wastes arising from winery [96]. Another approach that has been recently evaluated is vermicomposting, especifically of grape marc, although in this case the advantage arises from the physical processing of these residues, which allows to separate the material (vermicompost) from a residue that mainly contains grape seeds, thus facilitating the extraction of polyphenols from this latter organic material [100].

#### 4.2.2. UV Radiation

The usage of UV radiation has been gaining particular attention concerning the active improvement of the composition of these wastes, besides the separation of trapped or condensed compounds, and metabolites produced by microorganisms (generated in the plant material during storage as a consequence of the metabolism of the microorganisms naturally occurring in planta material). Nevertheless, regarding this approach, the impact of this technique relies on the response of the vegetal tissues’ defense mechanisms, reflected in the segregation of phytochemicals, thus, being suitable solely for live tissues when metabolic modifications are intended, or those possessing, at least, certain viable biochemical mechanisms [9]. Actually, the majority of the works undertaken, regarding the application of these techniques, deals with grapes, either separated, or entire bunches, while some works have been already developed for grape stems [6,101,102].

Moreover, in a somewhat different approach, the effect of postharvest application of UV-C radiation has been also assessed in conventional and organic grapes (*Vitis labrusca* cv. ‘Concord’), in a process that is closer to the treatment of residues, eventually occurring at the transformation step, since the grapes are arranged in a single layer on white trays, similarly to what can happen in the post-steeming step, being subsequently subjected to radiation in a cabin equipped with UV-C lamps. In this sense, Pinto and col. observed that UV-C radiation stimulated gene expression for the biosynthesis of phenolic compounds, leading to an accumulation of these metabolites, while these changes were distinct for organic and conventional grapes, being higher in the latter [101].

Another benefit to be obtained from this approach might be the possible improvement of the contents in stilbenes, which are also synthesized in response to external stresses, such as UV-C radiation, or even the ozone concentration, while these stresses are able to increase the stilbene levels up to several hundred-fold relative to untreated materials in grape skins and leaves [102]. Therefore, the application of these approaches during industrial processes undertaken to obtain must arises as a feasible possibility, increasing the concentration of these bioactive compounds not only in must, but also in the residue derived from the crushing procedure (grape pomace), allowing new valorization opportunities as improved sources of bioactive phytochemicals. Moreover, as recently reviewed by Teixeira and col., a plethora of works supports the notion that white and red grapes present distinct profiles concerning the presence of these compounds, being observed the presence of stilbene derivatives in stems, seeds, and pomaces from red varieties, while concerning white cultivars, these derivatives have been found solely in stems and skins [3].

Almost as a by-product of the previous treatments, the usage of UV radiation can also be used to effectively destroy harmful contaminants, such as ochratoxin-A, either in must or in by-products, so as to eliminate the microbiological agents responsible for the occurrence of toxins and the occurrence of unwanted parallel fermentation, thus, allowing the conduction of intended fermentation processes to improve the composition of the winery residues [10,95,96]. In fact, the photo-degradation of ochratoxin-A by UV radiation has been thoroughly assessed, with this molecule strongly absorbing radiation between 255 and 455 nm, while this interval has been shown to be also effective in reducing the microbial burden in must. Thus, it has been shown that ochratoxin-A is degraded by treatments with UV irradiation, following first-order kinetics, while the pH of the aqueous solutions influences the degree of photo-degradation of this molecule, with a greater degradation at pH 7 than in acidic medium (pH = 4). The same process in must is featured by lower kinetic constants, probably due to the global photo-protector effect of other substances present. Actually, this latter fact suggests that the absorption of UV radiation by the phytochemicals present—the purpose to which they were initially synthesized in plants—represents a pitfall of this treatment, protecting toxins and microbiota from radiation, and thus diminishing its efficiency [103].

According to the aforementioned facts, UV radiation treatments have to be taken into account regarding the improvement of the winery industry by-products to be further used for distinct purposes. Thus, those residues maintaining their metabolic activity, such as stems or shoots, present greater potential to be improved through radiation treatment, due to the effect of this radiation on the biochemical pathways responsible for plants response to stress, which lead to the synthesis of additional phytochemical compounds. Moreover, another approach to be considered relies on the post-harvest treatment of grapes or entire bunches, aiming to the profit foreseen either on wine quality, or from the effectiveness of this approach in the subsequent residues valorization.

#### 4.2.3. Liberation of Compounds through Electricity Pulses, and Thermal and Enzymatic Treatments

Although to date the obtimal extration conditions for bioactive phytochemicals using conventional analytical solvents have been widely reported, many times the outcomes are not appropriate for the practical application of these compounds towards new added value products, because the limited yields or toxic features of the obtained extracts. Thus, in order to overcome these constraints, the challenge of applying new pretreatment technologies contributing to reduce solvents consumption, temperatures and/or the extraction time, turning the extraction procedures more efficient and sustainable, has been pointed out pasionatelly in the recent years. In this connection, the application of pulse electric fields and high voltage electrical discharges has been deeply reviewed recently by Puertolas et al. who retrieved the most relevant evidence on their potential applications and advantages that can be summarized as improved extraction yield, increased extraction rate (decreasing extraction time), decreased intensity of conventional extraction parameters, and reduced energy cost of conventional extraction protocols [104].

When evaluating the specific application of this technology to winery by-products, it is only in the last decade that the efficiency of pulse electric fields in cells permeabilization has been demonstrated, likewise for the improvement of anthocyanidins extraction as a consequence of the augment in the plant cells’ permeability [105].

The electric treatments of plant materias addressed to improve the efficiency of the phytochemicals extraction consist basically on subjecting the target material to the intermittent application (<300 Hz) of electric fields of moderate−high intensity (0.1–20 kV/cm) and short duration, which causes the formation of micropores in cell membranes. However, the impact of these electric treatments on the efficiency of the extraction processes is not only dependent on the settings of the electric discharges but also on the physicochemical properties of the treated tissues and cells. As a result, it has been pointed out that depending on these factors the intensity of the electric treatments needs to range between 0.1 and 10.0 kV/cm, whilst they are not competent to raise the extraction efficiency in highly lignified plant materials for which intensities of up to 20.0 kV/cm are required [104]. However, this technology is not immune to drawbacks. Actually, recently it has been demonstrated that as a consequence of the electric flow some molecules can pass from the electrode to the plant materials, with consequences that nowadays are underexplored but that seem to limit the use of the extracts obtained. The identification of this situation has prompted to investigate new materials that limit the impact of deleterious contaminants [104].

In order to overcome the constraints enclosed to each extraction strategy, recent investigations have supported a joint contribution of the features of the electric pulses applied and additional extraction variables, taking into account the specific characteristics of the plant material under consideration [104]. This information allows to envisage that the most appropriate alternative to taking significant advantage of using this combined extraction strategy, though this requires to re-evaluate the settings for the classical extraction variables in the frame of applyling electric pulses in order to gain a further insight in the real scope and potential of this procedure towards environmental and economic advantages. Hence, according to Bousseta et al. pretreatment with high voltage electric discharges provide extraction advantages regarding efficiency and extraction-times relatively to pulse electric fields in aqueus medium [106], whilst the application of high voltage electric discharges pretreatment of winery residues, before hydroethanolic extraction, allows the maximum recovery of polyphenols [107]. However, to get a high yield is not the only feature of phytochemical extracts that should be considered, so, the application of pulsed electric fields allows one to obtain extracts with low turbidity, which is crucial for the proper development of the following stages devoted to purify individual compounds within polyphenolic extracts, thereby reducing the cost of the recovery process, whilst high voltage electric discharges can promote the release of other molecules that interfere in the subsequent purification processes [104]. Interestingly, the scale-up and practical application of these findings by the industry seems to be technically, energetically, and economically feasible, although strongly dependent on the physico-chemical characteriscis of the material to be processed, as well as on the chemical properties of the target compounds on which the extraction process would be focussed.

Another approach envisaged for the improvement of these residues relies on thermal treatments, which have been already assessed for grape stems and pomace [99], while such a methodology has been also previously evaluated concerning additional agro-food wastes, such as corncob or almond shells [108]. In the work developed by Chamorro and col., where grape seed products have been assessed concerning this treatment, it was observed that the total extractable polyphenols content, tannin content, procyanidin components, and the antioxidant activity were not affected by furnace thermal treatment of grape stems and pomace, while autoclave treatment caused an extensive hydrolysis of gallocatechin (70.0%), catechin (61.0%), epicatechin (65.0%), procyanidin B1 (75.0%) and procyanidin B2 (73.0%) in grape stems, besides an increase in gallic acid (71.0%), gallocatechin (100.0%), and epicatechin gallate (129.0%) in grape pomace. Thus, it was concluded that the effect of autoclave is more severe than furnace heat treatment, due to the modifications induced on the phenolic profile, which differ depending on the grape seed product used [99].

Besides the liberation of phytochemicals due to the degradation of the cell wall structure by microbial enzymes, produced during fermentation, another valuable approach presently envisaged relies on the direct application of such enzymes, in order to take benefit from these modifications, without delayed or lesser controlled fermentation processes. In this sense, Chamorro et al. have studied the changes taking place in the polyphenolic and polysaccharide contents of grape stems and pomace after enzymatic treatment, namely, by resorting to cellulase, tannase, and pectinase [98]. Generally, pectinolytic enzymes disintegrate the plant cell wall matrix of grape pomace, releasing monosaccharides and facilitating the polyphenol libertation, whilst a higher effect is obtained when both enzymes are combined [98].

Specifically, the main observations retrieved from that study were that the use of pectinase and tannase in grape by-products assisted the liberation and extraction of phenolic compounds, finally resulting in a higher antioxidant capacity of the extracts from these materials, respecting the untreated ones. Moreover, the usage of either tannase or pectinase in grape pomace, and tannase in grape stems, changed the galloylated form of catechin to its free form, releasing gallic acid. Therefore, these results support the potential of pectinase, tannase, and combination of enzymes to release polyphenols and monosaccharides from grape by-products, thus improving the antioxidant capacity and the nutritional value of these residues, while these enzyme polyphenol extraction processes might be applied to allow the bioactive compounds’ production from these residues [99].

Summarizing, the aforementioned solutions demand cooperative efforts from a range of actors and disciplines from engineering to (bio)chemistry, bio(techno)logy, environmental sciences, legislation, and economics, in order to achieve imaginative alternatives, hopefully leading towards the discovery of new promising bio-active compounds, besides the valorization of these residues, which could contribute to a more sustainable industrial sector and society [3,10].

## 5. Structure-Activity Relationship: What Do We Know?

### 5.1. Health Effects and Antioxidant Properties of Phenols

The vast majority of the health benefits attributed to the compounds present in the winery residues—similarly to those atributed to the wine consumption—have been traditionally assigned to their radical scavenging capacity, even though several biological functions displayed by these compounds, which have been lately assessed, are due to specific mechanisms of action no directly related with their radical scavenging power. In which respects to the antioxidant activity, its importance arises from the necessity to avoid the damage (or lower the severity of the pathophysiological processes associated) due to reactive oxygen species (ROS). These deleterious molecular species result from the conversion of molecular oxygen, in the process of sequential one-electron reductions, and occur constantly in living organisms without necessary exposure to any external harmful factors, though they are exacerbated due to these aggressions [95]. However, depending on the method of extraction of the grape residues, as well as on the grape variety, the extracts obtained can develop antioxidant or prooxidant activities. This situation needs further exploration in the near future, as well as the establishment of proper combinations with other compounds, aiming to the modulation of the prooxidant activity of extracts obtained under specific conditions [109], in order obtain polyphenolic combinations suitable to be used in the development of healthy products, avoiding deleterious reactions.

The formation of these reactive species may arise from any enzymatic oxidative complex, which may eventually release an amount of bound oxygen as ROS into the intracellular or extracellular spaces. Morevoer, there are some oxidases (e.g., neutrophil NADPH oxidase), for which ROS release represents the main function [110,111], while there are other non-enzymatic reactions, involving transition metal ions, which can lead to the formation of highly reactive hydroxyl radicals (HO^•^) from less reactive ROS (superoxide, OO^•−^ and hydrogen peroxide, H_2_O_2_), which otherwise display important roles respecting to physiological regulation and cellular signalling [112]. Furthermore, these highly reactive species can readily lead to the oxidation of biological molecules, thus starting a chain of events that can lead to oxidative stress, eventually resulting in severe adverse health effects, being widely accepted that free radical oxidation of membrane lipids, in the presence of molecular oxygen, represents one crucial stage in this process [95,113].

Therefore, in order to avoid these potentially critical damages induced by ROS, the selective pressure led the aerobic organisms to develop a complex defence system to deal with free radical oxidation, eliminating initiators and/or initiator producers, which include a number of ROS-metabolising enzymes (superoxide dismutases, glutathione peroxidases, catalase, thioredoxin peroxidases), besides metal ion sequestration proteins (transferrin, ceruloplasmin) [114]. Furthermore, the initiation or chain-carrying free radicals processes can be interrupted by a number of low molecular weight compounds, which can act either as preventives (urea, thiols, and ascorbic acid, among others) or chain-breaking (α-tocopherol, ubiquinol, and β-carotene, among others), while plant phenols and polyphenols, such as the ones to be found in the winery residues, can play both roles. Moreover, other agents, such as ascorbic acid and thiols can either present activity, or even participate in regeneration processes of other antioxidants [115].

Therefore, during the last decades the importance of these antioxidant molecules has drawn the attention of the research community to the phenolic antioxidants, mainly to those arising from natural sources, due to the number of beneficial health effects of several foods and foodstufs, related with the presence of these compounds, and the importance of biological membrane-bound phenolic antioxidants, such as α-tocopherol and ubiquinol. Moreover, some of the most important industrial and food antioxidants (butylated hydroxytoluene, butylated hydroxyanisole, and propyl gallate) are phenolic compounds, which are often derivatives or structurally similar to compounds occurring spontaneously in vegetal matrices [116,117], such as the residues resulting from the wine production [3]. Moreover, the plethora of natural plant phenolic compounds identified so far (>8000 [112]) represents a virtually limitless source for the developement of experimental and clinical research, resulting in a massive quantity of data for the computational approaches to take advantage of.

Concerning the direct antioxidant effects of phenolic compounds—the family comprising the majority of the compounds to be valorized concerning these residues—, these are determined by their reactions with either ROS or other radicals at the initiation step, or with peroxyl radicals at the propagation step [108], while these reactions can occur resorting to several mechanisms, mainly depending on the compounds’ structure and electronic delocalization, besides the solvent characteristics. These mechanisms are developed by four distinct processes, which have been fully described till the present date, namely, hydrogen atom transfer (HAT), single electron transfer (SET), and sequential proton loss-electron transfer (SPLET), besides the so-called radical adduct formation (RAF), these processes differing in which respects to rate-controlling steps and kinetic, though presenting similar overall balances of reactants and products [95,113].

As aforementioned, natural and synthetic phenols and polyphenols are responsible for a spectrum of additional biological activities, which are not directly related to the antioxidant activity. Indeed, some of these compounds interact with certain membrane transporters [118], or inhibit distinct protein kinases, such as catechol-*O*-methyltransferase, lipoxygenases, cyclooxygenases, xanthine oxidase, and membrane bound NADPH oxidase [119,120]. Another capability of these compounds, mainly those including a catechol or carboxylate group, is the scavenging of transition metal ions, which indirectly contributes to antioxidant activity, besides avoiding damage in biological molecules due to these metals [121]. Moreover, some of them may also act as in vivo prooxidants, depending on their concentration, by depressing the Keap1/Nrf2 signalling pathway, thus enhancing the expression of a number of ROS-scavenging enzymes [122], whereas these effects of phenols result in their indirect antioxidant action under in vivo conditions in the framework of complex biological systems, concomitantly to the ability to bind metal ions. Furthermore, other biological activities have been recentlyl assigned to phenolic compounds found in the winery industry residues (Table 1, Table 2, Table 3, Table 4 and Table 5), besides antioxidant, comprising: anti-inflammatory, anticancer, and cardiovascular protection activities [3,123], for which their specific mechanisms of action will be subsquently discussed in the present work.

### 5.2. Importance of H-Bond Interactions for Conformation and Function

**Figure 2:**
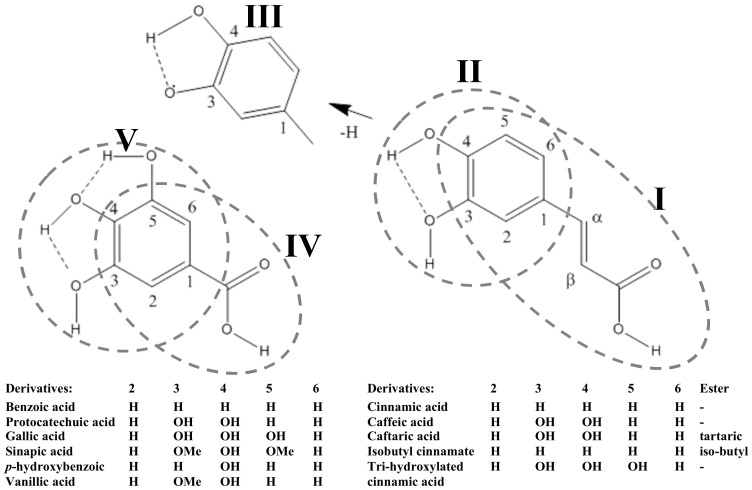
Representative structures for phenolic acids: (**I**) cinnamic moiety; (**II**) catechol moiety; (**III**) radical formed by catechol; (**IV**) benzoic acid moiety; (**V**) pyrogallol moiety. Substitution patterns for some derivatives are included.

The radical scavenging activity (which is an indicator of the capacity of bioactive compounds to modulate oxidative stress in biological systems) represents one of the most important characteristics, concerning the phytochemicals found in these residues, while the ability of some specific groups to be involved, or not, in H-bond interactions will greatly affect this feature. This occurs mainly because HAT is one of the most important mechanisms regarding this activity, whereas this kind of interaction will alter the dynamics of the H atom liberation [108]. Actually, when hydrogen-type interactions take place, the (O–H) covalent bond is affected, which is reflected in a lengthening and weakening of the original O–H bond as the hydrogen is attracted away from the donor toward the acceptor, being associated with the decrease of the O^…^O distance occurring simultaneosuly to the strengthening of the (O–H^…^O) hydrogen bond close contact (Figure 2). Actually, the interdependence of the *d*(O–H) and *d*(H^…^O) distances has been verified by neutron diffraction data, allowing to conclude that very strong H-bonds in a (O–H^…^O) system, tendentiously converge to the limit *d*(O–H)=*d*(H^…^O) ≈ 1.2 Å, for a *d*(O^…^O) distance of ca. 2.40 Å, thus showing the tendency for the (O-H) covalent to become weaker, whereas some of its electronic density is diverted to the (H^…^O) interaction [124,125].

Moreover, this kind of interaction may occur either at the intra or intermolecular level, potentially altering the conformational preferences in both cases. For instance, in different crystallization procedures, the same compound can display distinct conformations in the solid state, which is due to this kind of H-bond interaction, formed during the crystallization, and occur within the crystal lattice [124,125,126]. Moreover, those interactions occurring at the intra-molecular level can lead to the formation of additional cyclic structures, mainly in the case of flavonoids, for instance, the enolic –OH group in the position C-3 in flavones forms a relatively weak hydrogen bond with the neighbouring carbonyl group (Figure 2III), which increases planarity, and thus electronic delocalization, consequently increasing their ability to accomodate one unpaired electron with minimal fuss, thus facilitating the liberation of an H atom [126,127,128,129]. Furthermore, this effect is exacerbated when the C-3 position is occupied by a carboxylic group, with the spatial proximity between the H atom and the C4=O leading to a strong H-bond type interaction, and forming a rather stable 6-atom ring, as observed in the case of a 5-OH (Figure 2IV,V), which is reflected in the anomalous high pka presented by such a compound of ca. 8.85 [130].

Furthermore, the occurence of these interactions may directly influence the capacity to act as antioxidant, such as in the case of a molecule containing a catechol moiety, namely di-hydroxylated cinnamic or benzoic derivatives, which are widely present in winery residues, containing two contiguous hydroxyl groups, while the occurrence of such interaction facilitates the release of a H atom, since the remaining one will participate in a (O–H^…^O) interaction (Figure 2II,III), thus stabilizing the catechol system [125]. This occurs because the –O^•^ radical formed after H-atom abstraction has significant electron-withdrawing character, thus increasing the H-bond donating ability of the bound –OH [131], while the –O^•^ group itself is a much better H-bond acceptor than the hydroxyl group [125]. Moreover, reactive OH groups, such as H-bond acceptor catechol (Figure 2II) and pyrogallol (Figure 2V), the most common structural features in natural polyphenols presenting such interactions, act as chain-breaking antioxidants, being characterized by the presence of phenolic –OH groups acting both as H-bond donors (HBD) and acceptors (HBA). Actually, in those compounds possessing such a structural feature, the intramolecular H-bond is mantained during the reaction with peroxyl radicals, so that the ortho –OH group stabilizes the phenol, as well as the phenoxyl radical, due to the electron polarization in the radical [132].

**Figure 3:**
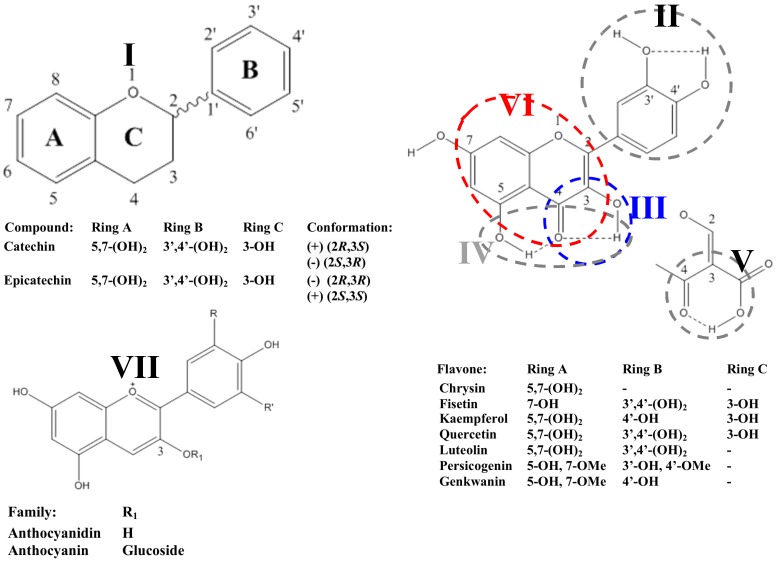
Representative structures for flavonoids: (**I**) flavonoid moiety; (**II**) catechol moiety; (**III**) representation of the intramolecular H-bond formed by 3-OH (Blue); (**IV**) representation of the simultaneous H-bonds formed by 3-OH and 5-OH (Grey); (**V**) representation of the six-atom extra ring formed due to a 3-COOH; (**VI**) chromone moiety (Red); (**VII**) generic representative structure for anthocyanins. Substitution patterns for some derivatives are included.

As a consequence of the contribution of this kind of interactions to enhance molecular stability, its occurrence, which is preferred respecting non-bonded conformations [129], can also contribute to the retention of the H-atom. For instance, concerning the flavonoids, 7-hydroxyflavone and chrysin (5,7-hydroxyflavone), the –OH group in position 5, in the latter, is blocked by the H-bond interaction with the neighbouring carbonyl (Figure 3), while these compounds display equivalent antioxidant activity, therefore, it can be elated that the functional moiety is the –OH in position 7 [125,132].

Moreover, the availability to liberate a H-atom, reflected in the bond dissociation energy BDE(O–H), of phenols, and therefore on their reactivity toward free radicals, can be influenced by intramolecular H-bonds not involving the “reactive” –OH group, since H-bond effects are relevant also in polyphenols having electron-withdrawing substituents, such as the aforementioned ones possessing a carbonyl group. Generally, the effects of remote H-bonding can either result in an enhancement or reduction of the BDE(OH), due to the modulation of the electron donor or electron-withdrawing character of substituents in conjugated position (commonly in *para*), concerning the most reactive –OH function, ultimately resulting in an increase or decrease of the antioxidant activity. Thus, these effects have to be taken into account, in order to racionalize the complex features underlying the reactivity of natural flavonoids, where remote H-bonding interactions occur alongside H-bonding to neighbouring (HBA or HBD) groups, in the same molecule. Actually, according to these facts, the same antioxidant activity observed for chrysin and 7-hydroxyflavone may be ascribed to the remote H-bonding of the *meta*–OH to the carbonyl group occurring in chrysin (Figure 3), being rather complex than a simple arrest of the H-atom by the existent intra-molecular bond [125,132,133].

In fact, quercetin, a very-well known antioxidant flavonoid, from which several derivatives are found in distinct winery industry residues (Figure 3; Table 3) presents most of the aforementioned structural features, thus, representing a good model to summarise all the distinct dynamics that may occur simultaneously concerning this kind of polyphenols, due to the intricate interplay between non-covalent interactions in this molecule. In this sense, the reactivity of the 7-OH is reduced respecting the same OH group in 7-hydroxyflavone, due to the remote H-bond effect exerted by the 5-OH, besides other groups [125,129]. On the other hand, the 3-OH in quercetin is expected to be more reactive than in 3-hydroxyflavone, since in quercetin the carbonyl group is also involved in a H-bond from the 5-OH (Figure 3IV), thus representing a relatively weaker HBA group, respecting the former compound [95,125,132].

Nevertheless, it has been suggested that the noticeable antioxidant behavior of quercetin arises mainly from the catechol moiety (Figure 3II), whose reactivity is increased by the H-bonding interaction between the two neighboring hydroxyl groups, whereas the A-aromatic ring (Figure 3I), may also present a rather important contribution to this activity, due to the substitution pattern observed in this molecule [132]. Finally, concerning these quercetin-like systems, another feature to be pointed, is the enhanced planarity due to the extended electronic delocalization arising from all the H-bond interactions and virtual ‘extra-rings’ formed (Figure 3II,IV), leading to an augmented ability to accommodate unpaired electrons, contributing to the stability of the radicals and consequently to the remarkable antioxidant activity observed [95,125].

Furthermore, besides these intramolecular H-bonds occurring within a molecule, the H-bond interactions that occur between molecules, either of the same compound, or from distinct compounds—which might be present in the same matrix, or even the solvent molecules—play a fundamental role, not only concerning the antioxidant activity, but also regarding specific mechanisms of action, while solvent effects might be crucial in both cases. For instance, historically advantage has been taken of the ability of polyphenols to bind proteins, in the usage of the former compounds as tanning agents for the conversion of animal skins into leather, while the same property is presently used to clarify wines from some excesses of polyphenols, resorting to proteins [134].

Therefore, several studies have been developed in order to assess the actual antioxidant activity of polyphenols in the presence of various biological molecules, since it was observed that, in some food matrices, the binding of flavonoids to proteins may lower their bioavailability and antioxidant activity in vivo, for instance, quercetin has been proposed to bind noncovalently to albumin, by forming H-bonds, which compromises their remarkable antioxidant activity [132,135]. Moreover, the intermolecular H-bonds are on the basis of the ability of some of these compounds to bind into distinct biological systems, not only to the proteic or enzymatic moiety, but also other important biological molecules, such as nucleic acids and lipids, being thus indispensible for the occurrence of several important activities [95,132].

Finally, in order to understand these aspects, it is important to rationalize the influence of non-covalent interactions on the reactivity of simpler phenols towards peroxyl (and other) radicals, or the solvent itself, which may ultimately be crucial for the participation of these compounds in the biological processes mentioned [132]. Actually, the assessment of the inhibition of the autoxidation of styrene by various phenols has shown that this effect decreased in the presence of polar solvents, being more pronounced with unhindered than with 2,6-di-*tert*-butyl-substituted phenols, as well as with acidic rather than non-acidic phenols, which has been assigned to the H-bond formation between the phenolic –OH group of the antioxidant and the solvent, acting as HBA, and thus competing with the initiating step radicals [132].

The impact of the solvent in these mechanisms is generally referred to as kinetic solvent effects, a theory based on the experimental finding that abstracting radicals are not significantly complexed by the solvent or that their intrinsic reactivity is not affected by this phenomenon [136]. Interestingly, the magnitude of this effect is possibly noticeable for good H-bond donors, like most natural polyphenols, with equilibrium rate constants for the liberation of a H-atom ranging over orders of magnitude for the same antioxidant in different media. Therefore, this effect has to be taken into account when the antioxidant performance of natural or synthetic compounds is evaluated under different settings, in order to realistically compare their potential. Moreover, the decrease in antioxidant activity, observed when polyphenols are non-covalently bound to biomolecules, can be explained on a similar basis, with these interactions playing the same role as those that are due to the solvent molecules [132].

Furthermore, deviations from the behavior expected from the “classical” kinetic solvent effects model have been registered. As an example, abnormally high rate constants, for the liberation of a H-atom, have been observed in alcohols, which were understood at the light of a different acid-base catalysis-like mechanism, consisting in a two-steps sequential proton-loss electron-transfer (SPLET). Even though this behavior has been generically observed for alcohols, also acidic H-bonded phenols, could transfer the proton to the solvent itself and subsquently undergo a fast electron transfer to an electron deficient radical, which ultimately results in an accelerated abstraction reaction [137]. In this connection, the flavonoids morin and quercetin have been reported to react with DPPH^•^ with rate constants about 1000-fold higher in methanol than in dioxane, which present equivalent HBA ability, due to the SPLET mechanism [134].

Moreover, the higher reaction rate in alcoholic (HBD) solvents was assigned to their H-bond with the oxygen of the alkoxyl radical, since such an interaction would become progressively stronger during the reaction progress, thus stabilizing the transition state, while this observation has been reinforced by the rate of reaction of hydroxyl radicals with a variety of hydrocarbons. This has been found to be larger, by as much as two orders of magnitude, in water than in polar aprotic solvents like acetonitrile. Therefore, it can be clearly pointed that deviations from the classical KSE model, which can be expected in protic solvents, are mainly due to the modulation of the reactivity of the abstracting radical, caused by H-bond donation to the (δ−) O–X moiety in the transition state [132,138].

Nevertheless, to date it has been not clearly understood if such mechanism plays a preponderant role concerning the reaction of antioxidants with peroxyl radicals, an important measure of the antioxidant potency of a compound at the intra-cellular level. Moreover, the acceleration occurring in reactions with ROO^•^, in the presence of bases, has been established, but only in specific cases, namely, for poly-hydroxylated antioxidants, such as caffeic acid, ascorbic acid, and 5-hydroxy-6-methyluracil, whereas it has been recently shown that, in the aforementioned cases, ROO^•^ can abstract a H-atom from the electron rich anion of the antioxidant [132].

Summarizing, while polyphenols represent the vast majority of the phytochemicals present in the winery industry residues (Table 1, Table 2, Table 3, Table 4 and Table 5) [3], with the antioxidant activity representing one of the most important features to be taken into account, regarding the valorization of wastes, structure-activity relationships have been clearly established, within a robust framework of kinetic and thermodynamic studies, solely for mono-phenolic antioxidants [132]. In this sense, natural polyphenolics have been rather less comprehensively understood, due to their more complex multifunctional structure, as well as the major role that non-covalent interactions play on their reactivity. Furthermore, in order to fully understand their antioxidant features, it is necessary to account with the interactions with the surrounding medium, such as the solvent, which is especially relevant for complex biological environments, where, besides water itself, a multitude of H-bonding ligands are available to modulate antioxidants’ reactivity [95,125,132].

Therefore, in order to evaluate all these preponderant features, concerning the activity of these compounds, molecular modelling arises as an indispensable tool for the assessment of structure and its impact on H-bonding, as well as H-bonding itself, besides the establishment of quantitative-structure activity relationships (QSARs), allowing to effectively relate the conformational preferences, with specific activities, such as antioxidant, amongst others.

### 5.3. Main Computational Methods in Studying Phenolic Antioxidants

Concomitantly to the increasing interest regarding the potential activities of natural phenolic antioxidants, the jointly usage of computational approaches for the optimization of processes and modelation of several properties of isolated molecules is claiming special attention. Therefore, the application of suitable computational methodologies has naturally gained its space, in which respects to the assessment and exploration of natural polyphenols, being presently applied to assess, not only structure (and conformation), but also its relationship with biological activities. Thus, the structure-activity relationships underlying these activities have become a hotpoint of strong research interest, with these computational methods providing new analytical chances to discern essential structural properties related to the antioxidant—or other—activities displayed by these compounds [95,132].

Moreover, concerning computational approaches, two main methodologies have to be stressed, namely: (i) ligand-based methods, which rely on the information about the bioactive compounds, and concerning their activities to be modelled; (ii) receptor- or structure-based methods, which are mainly based on data concerning the structure of the target biomacromolecule involved in a specific activity. Regarding these approaches, quantitative structure-activity relationships (QSAR) represent the methodology that ultimately allows to correlate the chemical structures within a series of compounds (defined by chemical descriptors) and their properties. This methodology, which represents the main category alongside 3D QSAR, allows the prediction of the biological activity, and other properties of new chemical structures, thus, providing useful information to rationalize the mechanisms of action within the series of compounds, concerning a concrete feature of interest, leading to the conception of an ideal structure regarding this specific effect/activity [95].

Nonetheless, in order to retrieve reliable data from this approach, the QSAR methodology comprises two indispensable steps, first, the development of the model, with resort to a training data set for which the features of interest have been previously assessed, being used for calibration purposes, and a second step, often referred to as validation. This latter stage relies on a statistical validation of the models developed, being undertaken with resort to data set(s) comprising samples that were not included in training, and represents a critical aspect of the QSAR process, which determines the reliability and the significance of the model, and thus, their ability to actually classify other molecules, concerning the feature of interest [139]. Finally, there are characteristics that are required for a quality QSAR model, besides robustness, such as simplicity, transparency, interpretability, and relevance [140].

In order to develop these QSAR models, concrete molecular descriptors have to be taken into account, for inclusion in QSAR models, since these are quantitative, while these descriptors can be chosen from a plethora of distinct options [141]. Generally, these are classified into distinct groups, with the electronic, hydrophobic, steric, constitutional, and topological descriptors, being amongst the most used designations. Moreover, these predictors can be either modeled, or experimentally assessed, for instance, steric and topological descriptors can be predicted in silico from the compounds **2D** or **3D** structural representation, resorting to quantum chemical (QC) calculations, while electronic and hydrophobic descriptors can also be obtained experimentally [95]. Nonetheless, concerning the modeling of antioxidant and radical scavenging properties of phenolic compounds, in QSAR approaches, the vast majority of the molecular descriptors used are the electronic ones, evaluated resorting to QC calculations, being often retrieved by semiempirical (MNDO, AM1, PM3) and ab initio Hartree-Fock methods, besides Density Functional Theory (DFT) [142].

Furthermore, all of these methods present known limitations, either concerning the determination of molecular geometries, as well as in the approximation of electronic properties [132]. In the former case, regarding the geometries, the presence of a negative vibrational frequency indicates a saddle-point in the potential energy surface, thus corresponding to a false minimum, and a non-existent conformation, while the energy differences between conformers, reflected in the relative population of each one, retrieve important clues concerning the actual conformation of a compound. In which respects to the electronic properties, the comparison between predicted features to the experimental ones, assessed in similar compounds (e.g., p*K*_a_, redox potential, and polarity), is necessary in order to ascertain the feasibility of a specific approach to produce reliable predictions for a specific chemical system [139].

Nowadays, calculations based on DFT represent the most widely used methods in computational research of phenolic antioxidants, as well as concerning other interesting chemical systems, due their reliability, and excellent quality/CPU time ratio, while the myriad of researchers applying these approaches for distinct chemical systems also provides a good comparison point for (virtually) any research work developed with resort to this kind of method [117,142].

Actually, even though being less computationally expensive than post-Hartree-Fock methods (such as MP2, based on Moller Plesset perturbation theory), DFT methods produce quite accurate structural geometries, mainly resorting to hybrid functionals (Hartree-Fock /DFT), such as B3LYP, when coupled to the Pople basis sets with polarization functions (e.g., 6-31G(d,p)), besides, the usage of extended basis sets, such as diffuse functions (6-31++G(d,p), also allows to retrieve satisfactory bond strengths, or even successfully mimic H-bonds, due to the extended molecular orbitals inserted by these basis sets. Moreover, other options, such as polarized double-zeta basis sets (e.g., cc-pVDZ) also allow obtaining remarkable results, while there are other functionals that have been developed specifically for the prediction of certain properties, such as the Voorhis and Scuseria’s kinetic-energy-dependent Exchange-orrelation DFT functional (VSXC), developed for the prediction of thermodynamic properties, presenting improved quality and suitability for these purposes [143,144].

Concerning the specific parameters to be modelled, these comprise: properties involving enthalpy-related reactions; the radical stability, and spin densities, the latter defining the probability of the unpaired electron to be found in a certain part of the molecule; structural features associated with radical scavenging by polyphenolic, as well as polycyclic compounds. Moreover, once assessed, the structural features—reflecting the impact of the substituents on conformation—can be directly used for the development of QSAR relating specific activities with certain substitution patterns, and difference between distinct molecules from the same family (e.g., number and position of OH substituents, presence of other functional groups and their position, number or aromatic groups) [125,145].

Furthermore, regarding the properties related to reaction-enthalpies, their importance arises mainly from the impact of these physico-chemical properties in bond-dissociation energy and ionisation potentials, while the description of alternative mechanisms of reactions, respecting hydrogen atom transfer, stimulated the modelation of theoretical parameters to be accounted within the sequencial electron transfer and sequencial proton-loss electron transfer approaches (proton dissociation enthalpies, proton affinity of phenoxide ion, and electron transfer enthalpies), which have recently gained visibility, concerning their importance for these simulations [95].

Finally, it is worth mentioning that some comprehensive reviews concerning structure-activity relationship analyses of antioxidants, have been published in the last decade, reinforcing the importance of these approaches [146,147,148], while there are also several publications available in the literature, regarding the assessment of QSAR, which include works developed resorting to directed syntesis, regarding some of the valuable compounds that can be found in the winery industry residues, which will be further discussed in the present work.

### 5.4. Presently Known SAR/QSAR for Compounds from Winery Industry

In which respects to the assessment of reliable structure-activity relationships for biological activities displayed by relevant compounds present in plant materials, and underexploited by the winery industry, the vast majority of the work developed so far deals with constituents from the wine itself, since these may display noticeable impact on the organoleptic and technological characterististics of this product. Meanwhile, concerning the compounds identified in the winery by-products, fewer assesments of biological activities have been developed, and thus, far less is known concerning the structure-activity relationships for the compounds to be found in these wastes. Nonetheless, several of the constituents found in wine, may be expected to be present in the by-products arising from their production, while some of these have also undergone the fermentation process (e.g., grape pomace, lees) [3].

Concerning phenolic acids, which are widely present in these residues, namely, grape leaves and pomace (Table 1), one of the main activities that have been recognized to these compounds concerns radical scavenging power, for which the general mechanisms have been previously discussed, while this activity is displayed by diverse compounds within the majority of the distinct families belonging to polyphenols, as well as other types of phytochemicals. Regarding phenolic acids (Table 1; Figure 2), besides the aforementioned importance of the hydroxyl groups number and position, and the H-bond dynamics, another important feature lies on the C***_α_***=C***_β_*** double bond existing in the cinnamic derivatives (Figure 2I), which creates enhanced electronic delocalization between the phenolic ring and carboxylic acid group, thus improving the stability of the formed radical, mainly when the aromatic moiety corresponds to a catechol group (Figure 2II), in the so-called caffeic acid derivatives, such as caftaric acid, besides other simpler derivatives [95,132].

In which respects to specific activities displayed by cinnamic derivatives, antimicrobial [149], anticancer [150], anti-inflammatory [150,151], and vasodilatory, represent some of the biological actions to be pointed to those compounds, while the latter activity, vasodilatory, has been assigned to both cinnamic and benzoic derivatives (Figure 2I,IV) [152]). Concerning the remarkable antimicrobial activity observed for cinnamic derivative, isobutyl cinnamate displayed a broad spectrum of activity against yeasts, besides Gram-positive and Gram-negative bacteria, with minimum inhibitory concentration values ranging between 43 and 12 μM, while this example illustrates how a simple substitution can have a significant impact on the biological properties of the molecules (e.g., isobutyl cinnamate vs. butyl cinnamate leading to a 4-fold minimum inhibitory concentration change against some microorganisms). For instance, fungal organisms are rather susceptible to the cinnamic aldehydes, while the bacteria are more affected by cinnamic acids, esters, and amides, with a noteworthy effect being observed for the cinnamic molecules against *Mycobacterium tuberculosis*, its growth being repeatedly inhibited by micromolar concentrations of compounds containing the cinnamic acid moiety (Figure 2I) [149].

In what respects to antioxidant, inflammatory, and anticancer activities, these are intrinsically related, while besides the preventive action regarding the last two pathological conditions, displayed by antioxidants, some cinnamic derivatives present inhibitory activity against enzymes that mediate the inflammatory process. For instance, trihydroxylated cinnamic acid (Figure 2) derivatives have been assessed regarding its inhibitory activity against the COX-2 enzyme, a mediator of the inflammatory response, besides representing an angiogenic factor in malignancies such as ovarian cancer. In this sense, it has been previously demonstrated that such compounds can inhibit ROS generating transcription factors closely linked to inflammation (e.g., necrosis factor-kappa-B). Concerning this effect, malonic acid, its ethyl ester, and a diethyl 2-(3,4,5-trihydroxy-phenylmethylene)malonate derivative have been assessed, in a work which observed an inhibitory activity of malonic acid similar to indomethacin, normally used as the standard drug, at the same molar concentration, while its ethyl ester presented a slightly lower activity [150]. Moreover, similar antioxidant activities have been registered for both compounds, leading to the assignment of this activity to presence of the pyrogallol moiety (Figure 2V). Therefore, the difference in the activity between compounds could be related, in some extent, to variations in the lipophilicity features due to the substitution pattern [150,151].

Concerning vasodilatory activity, attributed to both benzoic and cinnamic acid derivatives (Figure 2I,IV), a thorough study has been conducted by Mudnic et al. From this study it was revealed that the increase in the number of hydroxyl groups in the phenyl ring has a reverse relationship with the efficiency of the vasodilatory activity [152]. Hence, the monohydroxybenzoic acids, vanillic and mainly *p*-hydroxybenzoic acids, induce greater vasodilation than the dihydroxybenzoic and protocatechuic acids, while syringic and sinapic acids (Figure 2) have been described in this study as the most powerful compounds regarding the biological activity [152]. A reverse correlation between in vitro antioxidative and vasodilatory activities of the target phenolic acids was also observed, which is illustrated by the low vasodilatory activity of gallic acid (Figure 2), even though this compound was shown to be the strongest antioxidant among the traditionally tested phenolic acids. Furthermore, the QSAR study conducted to date described that the antioxidant and vasodilatory effects of phenolic acids, besides the number of hydroxyl groups in the phenyl ring, are also related to the degree of compactness and branching of molecules [152].

However, interesting biological activity is not the only feature conditioned by the chemical structure of phenolic acids. In addition to the functional properties, the bioavailability of these compounds is closely related with the chemical features of these compounds, whilst this biological characteristic condition the ultimate bio-activity expected from the compounds present in plant materials, traditionally studied as health promoters. In this connection, three-dimensional distributions of atomic polarisability of the tested molecules have been perceived as key features regulating the final concentration in cells after in vivo adminsitration. Moreover, concerning bioavailability, the drug transport properties, such as intestinal and oral absorption, as well as with blood–brain barrier penetration, have been shown to depend on the polar surface area, as well as lipophilicity and molar refractivity, through the QSAR studies undertaken [152].

**Figure 4:**
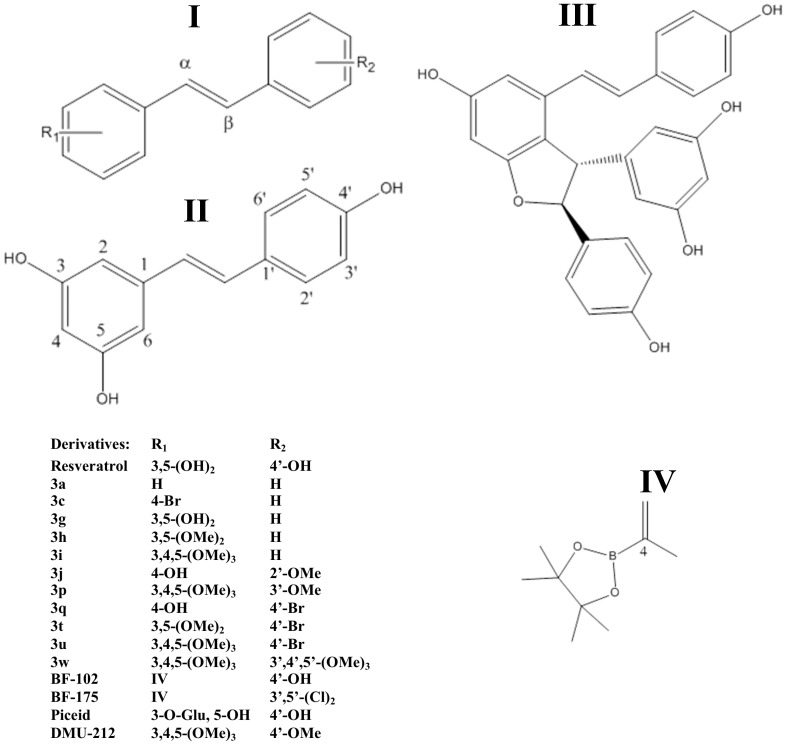
Representative structures for stilbenes: (**I**) basic *(E)*-stilbene structure, R_1_ and R_2_ are presented in order to indicate further substitution patterns; (**II**) representative structure for *trans*-resveratrol substitution pattern, indicating numbering; (**III**) *ε*-viniferin; (**IV**) 4-(4,4,5,5-tetramethyl-1,3,2-dioxaborolane) substituent group. Substitution patterns for some derivatives are included.

In which respect to the content in non-flavonoid compounds, stilbenes (Table 5, Figure 4) constitute and additional class to be considered, concerning valuable bioactive compounds in winery residues. Indeed, stilbenes exhibit a broad range of activities, either as individual compounds or in the form of purified extracts with a mixture of stilbenes (Figure 4I), while resveratrol (Figure 4II) represents one of the most well-charcterized compounds within this subclass, with several health benefits being assigned to this molecule, such as antioxidant, anticancer, antidiabetic, neuroprotective and anti-aging activities, besides cardioprotective capacity [153]. Moreover, important biological activities have been also assigned to other stilbenes found in these wastes, such as piceid, a glucosilated derivative from resveratrol (Figure 4), and *ε*-viniferin (Figure 4III), which results from the condensation of resveratrol and *trans*-piceid (Table 5) [41,154].

Regarding the biological activity of stilbenes, it has been pointed that the intake of a grape-derived polyphenolic extract containing stilbenes (0.4 mg/g *trans*-resveratrol and 0.9 mg/g *trans*-piceid), amongst other compounds, reduces the ratio of tumor growth by inhibiting angiogenesis and inducing apoptosis of malignant cells [155]. Additionally, certain stilbenes such as resveratrol, and glucosilated derivatives, namely, piceid and 2,3,5,4′-tetrahydrostilbene-2-*O*-d-glucoside (Figure 4) have been described as potent reducers of lipid levels, besides dispaying important activity as in vitro inhibitors of lung and colon cancer cells growth (at the micromolar scale), attenuating cells growth and metastatic events after oral administration [154].

Furthermore, since stilbenes have been promoted as reducers of systemic lipid levels in human, Das and col. have synthesized a series of pinacolyl boronate-substituted stilbenes, in order to further understand the role of these compounds as lipogenic inhibitors. Among these, the derivatives BF102 and BF175 evaluated in the referred work, presented noticeable lipogenesis inhibitory effect by suppressing the expression responsible for this activity in mammalian hepatocytes [156]. In addition, the former, also was able to inhibit the biosynthesis of both palmitate and cholesterol by suppressing *HMG-CoA* reductase gene expression. Thus, since sterol regulatory element binding proteins (SREBP) transcription factors are key activators of these genes, BF102 has been suggested as functioning through interference with the function of the SREBP system without significant toxic effects [156], although the specific mechanism(s) of action was not fully clarified in this study.

Concerning resveratrol (Figure 4II), a broader knowledge is available regarding the activities and mechanisms of action relative to other derivatives from these residues. For instance, this compound promotes vasodilatation through multiple mechanisms, mainly the stimulation of Ca^2+^/K^+^ activated channels and the enhancement of nitric oxide signalling in the endothelium, thus exerting vaso-relaxant activity [157,158]. Moreover, it has been described that resveratrol could delay tumor development through distinct complementary mechanisms, such as the inhibition of both forms of the cyclooxygenase enzyme, thus reducing the risk of developing many cancers, or by the induction of cell cycle arrest and apoptosis in tumor cells [159].

Furthermore, interestingly, this compound is capable of penetrating the blood–brain barrier, and exert strong neuroprotective effects, even at low doses, combating the neuronal dysfunction responsible for the symptoms described in Huntington’s and Alzheimer’s diseases, through the SIRT1 pathway [159]. Actually, this ability to activate proteins of the sirtuin family—especially SIRT1—represents one of the most intriguing observations, concerning the linkage between resveratrol and longevity, since the activity of this evolutionarily conserved family of deacetylases and ADP-ribosyltransferases was shown to be NAD^+^-dependent [160], hence, linking the activity of crucial regulatory factors to cellular energy levels. Furthemore, other activities, such as promotion of fat mobilization and lipolysis, or by down-regulation of inflammatory mediators, have been recognized to be SIRT1-dependent [154], and thus susceptible to be modulated by resveratrol.

Nonetheless, not all the activities of resveratrol arise from its capacity to modulate the SIRT1 system. For instance, this compound reduced atheromatous plaque formation in a region specific fashion, in a process occurring without detectable activation of SIRT1 or alterations in amyloid precursor protein processing, while changes have been observed in brain glutathione, which declined 21.0%, and brain cysteine, increasing 54.0% [154]. Moreover, even though SIRT1 is involved in some inflammatory mediators, many others, modulated by resveratrol, are active through independent pathways, such as the cAMP-PKA-AMPKA cascade or PI 3-K, which can function upstream of SIRT1 [161,162].

Concerning the effects of resveratrol on vasculature, the direct effects of this compound on endothelial cells comprise the stimulation of endothelial nitrogen oxygen synthase (eNOS) and vascular endothelial growth factor (VEGF) production and down-regulation of endothelin-1 [163], whereas VEGF production along with thioredoxin-1, and heme oxygenase-1 have been also shown to underlie resveratrol enhancement of neovascularization [164] alongside up-regulation by RES of VEGF receptor-2 (Flk-1), and, eNOS and induction of nitrogen oxygen synthase (iNOS) [75,165]. Furthermore, this compound rapidly activates estrogen receptors ‘a’ and ‘b’ (ERa and ERb), as well as Mitogen-activated Protein Kinase (MAPK), ultimately leading to atheroprotective responses [166]. Generically, besides these specific effects, the benefits of resveratrol concerning the vascular health arise from the blend of antioxidant, anti-inflammatory, anticoagulant, and fat-lowering activities [166].

In general, one relevant drawback limiting the maximum harnessing of the biological activity of these compounds is related to their bioavailability. Since the low oral bioavailability and metabolic stability of resveratrol limits its application, many clinical studies are presently focused on the corresponding pharmacokinetics [167,168], with several research works being presently in development. These studies have already identified organic metabolites, which biological potential is gaining interest in the last years [169]. Therefore, besides the previously mentioned derivatives with ‘fat lowering activity’, other compounds resulting from directed synthesis (Figure 4) are being assessed regarding cytotoxic and antiproliferative activities against malignant cells to prove, beyond reasonable doubts, the impact on the biological activities that some structural modifications can comprise, which would allow to take advantage from nature, concerning the power of bioactive compounds. In this sense, Zhang and col. tested the activity of 2-hydroxylated (*E*)-stilbenes against four different human cancer cell lines (Colo-205, MDA-468, HT29, and MGC80-3), while amongst the newly assessed compounds, the so-called 3p and 3t derivatives (Figure 4) exhibited much higher activities than resveratrol, with broad spectrum of activities [164,170]. Furthermore, *trans*-3,4,4′,5-tetramethoxystilbene (DMU-212), obtained from the methoxylation of the phenol groups, displayed improved pharmacokinetic properties, as compared to resveratrol, besides antiproliferation activity in various cancer cells [168].

In which respects to the impact of the structural modifications on biological activities of these 2-hydroxylated derivatives, the substitution with a H-atom in the position R2 (Figure 4I), in the 2-hydroxylated (*E*)-stilbene derivatives (**3a**–**3i**), led to a slightly reduction of the activity spectrum, exception made for the bromo and 3,5-dimethoxy- substituted derivatives **3c** and **3h**, respectively, presenting high antiproliferative activities against distinct tumoral cell lines [170]. On the other hand, when the R2-group was 2-OMe, the resulting compounds **3j**–**3n** present selective high activities against a narrow spectrum of antiproliferative activities, while when the same position (R1) is occupied by 3-OMe, the corresponding compounds **3o** and **3p** display higher activities, especially when the R1-group is 3,4,5-(OMe)_3_. Actually, the highest antiproliferative activity, registered for **3p**, has been observed for stomach cancer cell line (IC_50_ = 35 nM) [170].

Finally, when the R2-group is constituted by 4′-Br (compounds **3q**–**3u**), the target compound is found to display a broad spectrum of activities, while the derivatives with a 4′-NO_2_ substitution at R2, (compounds **3v** and **3w**) present activity solely against few tumor cell lines. Moreover, the wide spectrum of activities observed for the compounds **3a**–**3w** has suggested a close dependency of the biological potential on the nature of the substituent groups. Thus, methoxy and bromo seem to be the most interesting substituents to enhance the activity of 2-hydroxylated (*E*)-stilbenes [170]. Considering methoxylation, which also led to the obtainment of DMU-212 (Figure 4), a potential anti-angiogenic agent, the activity of this compound arises from the inhibition of the growth factor receptor-2 (VEGFR2) phosphorylation, thus acting as suppressor of the signaling pathways mediated by VEGFR2, inducing apoptosis in endothelial cells [168]. These research works have also shown the relevance of assessing the panel of possible synthetic derivatives, in order to understand the structural features underlying the biological activities of phytochemicals, such as resveratrol [154,168,170].

With respect to flavonoids (Figure 3I), as mentioned before, three main groups can be considered within winery by-products, namely, flavanols, flavonols, and anthocyanins (Table 2, Table 3 and Table 4). Since radical scavenging represents one of the main activities recognized to these compounds, this effect was the first to be the target for the development of thorough SARs. For instance, in 2007, Amic et al. have published a comprehensive work concerning the known SARs for the scavenging activity of flavonoids. In this work, where effective QSAR were established, it was pointed that the most active flavonoids possess a 3′,4′-dihydroxy substituted B ring (Figure 3II), corresponding to a catechol moiety, and/or 3-OH group, while the C2=C3 double bond conjugated with a 4-keto group, responsible for electron delocalization from the B ring, further enhances the radical-scavenging capacity (Figure 2VI) [147]. Also, the presence of both 3-OH and 5-OH groups in combination with a 4-carbonyl function (Figure 3IV) and a C2=C3 double bond, increases the radical scavenging activity in the absence of the 3′,4′-dihydroxy structure in the B ring. In these cases, the hydroxyl substituents in the chromone moeity (Figure 3VI), forming an extended catechol-like structure, due to the H-bonds, turns into a larger determinant of flavonoid antiradical activity [95,146].

Moreover, these works have been extended recently, mainly taking advantage of QC approaches, these methodologies being used, not only to assess the structural features underlying these molecules, but also to evaluate other relevant parameters, such as bond-dissociation energy or spin densities, to be further correlated with specific activities. Actually, in a work dealing with chromones, flavones and isoflavones (Figure 3), resorting to QC, where these parameters have been assessed, the distribution of the spin densities, as well as the energy necessary for the formation of each radical, have pointed the importance of planarity for the antioxidant activity of these compounds, which is enhanced by certain structural features that were identified. For instance, besides the presence of a catechol group located at the C-2 position (Figure 3II), also the number of hydroxyl substituents and their location in the molecule (preferably at C-3, C-5 and/or C-7; Figure 3), are determinant structural factors for their ability to scavenge free radicals. Moreover, the importance of C-7, which has not been previously shown, was demonstrated, since a hydroxyl group at this position, although not representing the most suitable site for the formation of the radical, renders extended electronic delocalization, in an effect which is incremented by the presence of a 5-OH, as observed in the cases of quercetin and kaempferol, and the majority of the flavones found in nature (Figure 3) [125,129,132].

Actually, the antioxidant activity was found to decrease according to the order quercetin > luteolin > fisetin > kaempferol, with the difference between luteolin and fisetin consisting on a change between a 5-OH by 3-OH substituent (Figure 3IV), thus showing the importance of the presence of the 5-OH group in these chemical systems—forming an H-bond with the carbonyl group—rendering luteolin more reactive against radicals than fisetin, which is a flavonol, even though the latter group is generally related to a remarkable antioxidant activity. Meanwhile, kaempferol, presenting 3, 5 and 7-OH substitution (Figure 3), represented the most active derivative without catechol substitution in B-ring [125].

Another interesting study envisaging the assessment of specific structure-activity relationship, considering the regulation of vascular endothelial genes by flavonoids, was developed by Martínez-Fernández and co-workers, in 2016, aiming to the description of structure-expression relationship of flavonoids regarding KLF-2 up-regulation. In this study, several flavonoids such as the ones found in the winery residues (e.g., quercetin, kaempferol, catechin, and epicatechin) (Table 2 and Table 3) have been assessed, while distinct structural features were evaluated concerning its influence for the ability to affect eNOS and endothelin-1 (ET-1) expression, in order to establish the structural basis of their bioactivity [145,171].

In the aforementioned work, developed by Martínez-Fernández et al., it has been observed that the most effective flavonoids in KLF-2 up-regulation (luteolin, genistein, rhoifolin, epicatechin, catechin, and apigenin) ultimately resulted in the highest values for eNOS expression, thus supporting the possibility that the increment of eNOS expression occurs through KLF-2 induction, while the relative effect of the different substructures on the up-regulation of eNOS mRNA expression followed the order: 3-*O*-glycosylation > double bond C2=C3 ≈ 3-hydroxylation > 4′-*O*-methoxylation > 3′-hydroxylation ≈ 4-carbonyl group > 7-rutinose glycosylation (Figure 2). On the other side, the features affecting the down-regulation of ET-1 mRNA production include the following: double bond C2=C3 ≈ 7-rutinose glycosylation > 4-carbonyl group > 7-*O*-neohesperidoside glycosylation, with the flavanones naringenin, hesperetin, and naringin, presenting the greatest reduction effect on the ET-1 mRNA expression [145], whereas this effect has been also assigned to fisetin, a flavonol (Figure 3III) [166].

Summarizing, the structure-activity relationship found for these compounds have shown that the most important elements concerning the regulation of eNOS expression were represented by: glycosylation and hydroxylation of C-ring, C2=C3 double bond in the C-ring, methoxylation and hydroxylation of the B-ring, ketone group in C4 in the C-ring, and glycosylation in C7 of the A-ring. Moreover, the most important features, concerning the reduction of vasoconstrictor ET-1 expression, are the C2=C3 double bond forming a benzopyran moiety, the glycosylation at C7 of the A-ring and the presence of a ketone group at C4, transforming the benzopyran into a chromonic skeleton (Figure 3VI) [145].

Another study, concerning the assessment of QSAR, was developed regarding the role of flavonoid analogues as inhibitors of p56^lck^ protein tyrosine kinase, an enzyme belonging to a class of important mediators of normal cellular signal transduction. In this work, the most important descriptors to be correlated with the ability of the inhibitors for binding to the enzyme active site were revealed, being represented by the maximal total interaction of the C-O bond of the chromone ring (C4=O) (Figure 3VI) and internal flexibility of the molecules. Moreover, correlations with the inhibitory activity have been observed for several descriptors related to electrostatic properties of the inhibitors, such as the average nucleophilic reactivity, the minimum Coulombic interaction, or the maximal antibonding contribution, besides the weighted negative surface area. The importance of electrostatic and quantum chemical descriptors for the interaction of flavonoids with the specific p56^lck^ enzymatic active site environment has been demonstrated by the results, while the maximal total interaction for a C–O bond was observed to be the most important factor in regression, thus, being strongly correlated with this specific inhibitory activity (*R*^2^ = 0.6379) [172].

Furthermore, SARs have been obtained for flavonoids on procoagulant activity in human monocytes stimulated by lipopolysaccharide, concerning the tissue factor, which is a 47-kD membrane-bound glycoprotein, and the primary initiator of the coagulation cascade in both haemostasis and thrombosis [173]. In this work, twelve representative natural compounds, including (+)-catechin and (−)-epicatechin, kaempferol and quercetin, have been assessed, while the latter compound, which is widely spread amongst the distinct winery residues (Figure 2, Table 3), appears to be the most prominent suppressor of tissue factor induction [174].

In the aforementioned work, dose-dependent inhibitory activities have been observed for eight compounds, presenting the following potency order: luteolin-7,3′,4′-trimethylether > 3′-hydroxygenkwanin > kaempferol > (−)-epicatechin > persicogenin > quercetin > 5-hydroxy-7,4′-dimethoxyflavone > genkwanin; while no obvious effects on tissue factor activity have been observed for the other four compouds, including (−)-epicatechin. The data registered allowed the establishment of SARs, while this analysis has clearly shown that methoxyl substituents in the A and B rings played a key role on the inhibitory activity, while a hydroxyl group at C-3 (Figure 3III) was also shown to be rather influent. On the other side, data available in the literature indicate that the presence of the C2=C3 unsaturated bond, conjugated with the C4=O functionality (Figure 3), did not present a visible influence, concerning this activity, whereas the presence of a glycosylated group significantly reduced the inhibitory effects [174].

Finally, anthocyanidins (Figure 3VII, Table 4), besides constituting a group of important compounds present in wine, also represent a diversity of constituents to be found, and potentially explored, in the winery residues, while glycosylation, which generally occurs at C-3 [3], leading to the formation of the so-called anthocyanins, represents the most important structural feature regarding the potential activities displayed by these compounds. In this sense, it has been recently published a revision paper, compiling the works which have previously assessed the impact of glycosylation in the biological activities of this family of compounds. Actually, glycosylation represents an essential step of anthocyanin formation in vivo, as well as the prerequisite for further modifications of anthocyanins, such as secondary glycosylation and acylation. Concerning the effects of this transformation, it has been widely established that anthocyanidin glycosylation (Figure 3VII) generally enhances the stability, though decreasing the bioavailability and anticancer activity, besides influencing the antioxidant activity in distinct manners, depending on the glycosylation site and the type and number of the glycosyl [175].

Even though several SARs, regarding glycosylation, have been uncovered over the last decades, detailed SARs attributable to the glycosylation are not entirely clear, while three main issues, concerning the understanding of this transformation are awaiting further study, namely: (i) the relationships between the glycosylation site and the bioavailability or the anticancer activity; (ii) the relation between the glycosylation and the antioxidant activity, which has been blurred by divergent results among different experiments; (iii) in sucessive glycosilations by two or more residues in the same site, the corresponding sugar chain per se can represent physiologically active oligosaccharides, which display specific bioactivity, possibly interacting with that of the anthocyanidins [176].

In order to tackle these shortcomings, more studies will have to be developed regarding anticancer activity, while for the second—concerning antioxidant activity—the establishment of reasonable and representative in vivo and in vitro experimental setups, resorting to standardized universally accepted methods, would contribute to mitigate this issue. Moreover, recent developments have pointed that the nature of the sugar residue may be crucial for the glycosides’ activity, presenting noticeable impact on the pharmacokinetic parameters of the substituted molecules [67,177]. In this sense, the type, quantity, and sequence of the sugar residues comprising the sugar chain, as well as bonding type, would have to be taken into consideration, regarding the establishment of comprehensive and reliable SARs of anthocyanidin glycosylation [176].

Summarizing, in what respects the importance of distinct functional groups for the specific roles of these compounds, the importance of several features has been established for some of these activities, while the impact in antioxidant activity represents the most well understood effect till the date. For instance, in phenolic acids (Figure 2, Table 1) the number of OH substituents has been clearly correlated with this activity, with gallic acid being the most powerful antioxidant amongst benzoic derivatives, while concerning the cinnamic derivatives, the C***_α_***=C***_β_*** double bond (Figure 2I), existent in these derivatives increases the electronic delocalization between the phenolic ring and carboxylic acid group, thus improving the stability of the radical, mainly when the aromatic moiety corresponds to a catechol group—displaying the ability to form a H-bond stabilized radical (Figure 2III) [95,132]. Furthermore, concerning cinnamic derivatives (Figure 2I), those molecules possessing such a moiety displayed noticeable activity against the presence of *Mycobacterium tuberculosis* [149].

For the other major group of phenolics found in these residues, flavonoids (Figure 3I, Table 2, Table 3 and Table 4), some studies envisaging structure-activity relationship have been also developed, mainly concerning the value of structural features occurring in nature (C2=C3 double bond, C4=O carbonyl, number and position of hydroxyl substituents (mainly at C3—comprising flavonols; Figure 3III), presence of catechol moiety at ring-B (Figure 3II), and glycolisation—generally at C3). Regarding the evaluation of each individual feature, the inclusion of the C2=C3 double bond in the C-ring was the second most important element (Figure 3VI), concerning the up-regulation of eNOS mRNA expression, and the most important feature, concerning the reduction of vasoconstrictor ET-1 expression, while this feature, when conjugated with C4=O group, is responsible for extended electronic delocalization from the B ring, further enhancing the radical-scavenging capacity [67,125,146]. Moreover, the C4=O group (Figure 3VI), by itself, represented the most important descriptor to be correlated with the ability of flavonoid analogues as inhibitors of p56^lck^ protein tyrosine kinase [172].

It has also to be mentioned that a catechol moiety in the place of the B-ring (Figure 3II) represents the most preponderant feature for the scavenging activity of flavonoids, due to the formation of the H-bond stabilized radicals, as observed for the di-hydroxylated cinnamic derivatives (Figure 2I). Moreover, hydroxylation, either in the B-ring, or in the A and C moieties is also very important for the biological activity, with those compounds displaying OH substitution in position 3, which distinguishes flavonols, forming a relatively weak hydrogen bond with the neighboring carboxyl group (Figure 3II) that increases planarity, and thus, radical stability [93,132]. Furthermore, the presence of both 3-OH and 5-OH, in combination with C4=O function (forming extended H-bond interactions; Figure 3IV) conjugated with C2=C3 double bond, further increases the radical scavenging activity in molecules lacking the B-catechol moiety. Moreover, 5-OH presence renders extended electronic delocalization, which alongside the presence of a 7-OH, makes this latter site suitable for the formation of a stable radical. Actually, kaempferol, presenting 3, 5 and 7-OH substituents, represents the most active derivative without a catechol group (Figure 3) [125,132], thus, showing that these are determinant structural features for the flavonoids ability to scavenge free radicals. Furthermore, hydroxylation of C-ring, alongside glycosylation, represent the most important chemical features, concerning the regulation of eNOS expression, while the latter substitution (glycosylation), significantly reduces the inhibitory effects for tissue factors, activity for which the introduction of methoxyl substituents in the and A and B ring (Figure 3) plays a key role [145,171].

Furthermore, concerning stilbenes—with resveratrol representing the most well known compound—the introduction of a sugar residue also plays a noticeable role, with glycosilated derivatives being pointed as reducers of lipid levels, besides displaying important activity as in vitro inhibitors of cancer cells growth, while methoxylated derivatives presented noticeable anti-angiogenic and anti-cancer activities (Figure 4) [154,170,171].

Regarding anthocyanidins, which are transformed into anthocyanins through glycosilation (Figure 3VII), the effect of such a transformation has been clearly related to molecular stability, also regulating the ulterior substitutions that can occur on the glycosilated compound, while the sugar and bond type are also preponderant for the activities displayed. Nevertheless, additional studies are necessary to understand the true impact of these modifications in this family of compounds [121,178].

## 6. Conclusions and Future Prospects

The technological breakthroughs achieved during the last decade have revolutionized the scope of utilization of natural products. The actual advance through this issue involves new developments concerning analytical tools (sensitivity and resolution, miniaturization, and high-throughput screening), molecular biology, computerization, and databases. Although the present review explores a very small part of the existing bio- and molecular diversity (concerning winery residues), from the information retrieved it is evident that joint interpretation of the current analytical and bioassay technologies will contribute, in the near future, to speed-up the assessment of molecular diversity and to significantly increase the effectiveness of lead-compounds discovery from Nature.

In the diversity of winery residues to take advantage from, namely, stems, pomace, and lees, distinct families of compounds comprising several molecules with enough potential as to be used for technological purposes or drug discovery can be found. Although these compounds have been extensively characterized so far, concerning their chemical properties and biological activities, additional efforts are still required to further understand the specific chemical traits behind desired functionalities. To gain this essential knowledge, which will allow the scientific community to move forward towards the achievement of actually powerful compounds with real application in functional products devoted to improve the population health.

At this point, the limiting factors, which remain to be overcome in the future, are those related to the capacity to optimize the processes used to isolate high purity compounds, and to develop the chemical procedures (synthesis) to obtain highly bioavailable and functional compounds, as well as the restricted concentrations of valuable compounds found in plant materials.

In connection with the current constraints on taking advantage of the bioactive compounds present in plant materials, several studies have been already developed (or in course), dealing with the improvement of these matrices, thus aiming to the sustainable and improved recovery of these compounds (in terms of purity and yield) by applying efficient physical, chemical, microbiological or enzymatic treatments. This advance will allow, in the near future, additional progress towards the obtainment of compounds with specific (desired) bioavailability and/or biofunctional properties.

Since the rational application of bioactive compounds present in plant material requires a full characterization of their chemical features, this knowledge is generating vast information on the chemical properties related to enhanced biological properties of interest. Hence, the current state-of-the-art requires a pragmatic application of the previously generated knowledge towards the development of new compounds, or through modification of those obtained from natural resources envisaging new species, which could respond to the current functional (biomedical or technonolgical) demands. However, this essential advance has to be based in a thorough understanding of the mechanisms of action observed, which is still inadequate to date, besides their involvement in intra-cellular biochemical pathways.

Actually, even though several works have been developed, regarding the isolation of substituted derivatives aiming to the evaluation of the impact of particular stuctural features on the activities, insufficient data are presently available concerning specific mechanisms of action. In this sense, the application of QC for the assessment of specific molecular descriptors, as well as the introduction of SAR and QSAR methodologies, will allow, not only the conception of improved structures, but also the disclosure of important biochemical processes, through which these activities occur, thus leading to a rational design of optimal compounds.

The joint application of the current knowledge, obtained from the separate disciplines involved in the valorization of natural resources, and the application of bioactive compounds towards obtaining valuable functional foods, will contribute in the near future to a more sustainable (circular) economy and to give response to the health claims of the world society.

## Figures and Tables

**Table 1:** Phenolic acids present in winery residues.

Compound	Concentration (microg·g^−1^ dw)				Analytical Approach ^Z^	Reference
	Skins	Seeds	Stems	Marc		
*Hydroxybenzoic acids*						
Gallic acid	-	-	-	82.00–99.00	LC-MS/MS	[29]
	2.20–82.00	6.50–224.00	70.40–469.00	10.50–459.00	HPLC-DAD	[2,27,30,31,32,33,34,35,36,37,38]
	0.38–0.74	-	-	-	HPLC-DAD-ESi-MSn	[24,39]
Gentisic acid	1.10–1.61	-	-	-	HPLC-DAD-ESi-MSn	[39]
Hydroxybenzoic acid	0.02–0.43	-	-		HPLC-DAD-ESi-MSn	[39]
Protocatechuic acid	-	-	-	4.10–5.00	LC-MS/MS	[29]
	6.00–13.00	2.00–10.00	66.00–115.00	0.50–4.20	HPLC-DAD	[27,38]
	0.08–0.13	-	-	-	HPLC-DAD-ESi-MSn	[24,39]
Syringic acid	1.60–3.30	-	-	-	HPLC-DAD	[31,35]
Vanillic acid	3.00–7.10	-	-	8.40–154.20	HPLC-DAD	[27,31,35,37]
*Hydroxycinnamic acids*						
Caffeic acid	1.00–21.00	2.00–9.00	5.00–40.00	-	HPLC-DAD	[38]
	0.15–2.72	-	-	-	HPLC-DAD-ESi-MSn	[39,40]
Caftaric acid	-	-	-	21.00–33.00	LC-MS/MS	[29]
	0.60–24.00	2.00–11.00	15.10–274.00	2.60–277.20	HPLC-DAD	[27,31,36,38,41]
	-	11.80–40.40	8.20–70.60	0.20–1.60	HPLC-DAD-ESi-MSn	[24,42]
Chlorogenic acid	0.29–0.32	-	-	-	HPLC-DAD-ESi-MSn	[40]
	40.50–231.10	28.70–68.00	-	-	HPLC-DAD	[43]
*trans*-Coutaric acid	0.10–5.50	0.40–7.50	6.90–20.70	6.10–63.40	HPLC-DAD	[36,38,44]
Fertaric acid	-	-	-	4.80–5.90	HPLC-DAD	[36]
Ferulic acid	-	-	-	6.70–27.80	HPLC-DAD	[37]
	0.23–3.65	-	-	-	HPLC-DAD-ESi-MSn	[39]

^Z^ HPLC-MS/MS, High Performance Liquid Chromatography coupled to Mass Spectrometer; HPLC-DAD, High Performance Liquid Chromatography coupled to Diode Array Detector; HPLC-DAD-ESi-MSn, High Performance Liquid Chromatography coupled to Diode Array Detector, Electro Spray Ionization, and Mass Spectrometer.

**Table 2:** Flavanols, hydrolysable tannins, and condensed tannins present in winery residues.

Compound	Concentration (microg·g^−1^ dw)				Analytical Approach ^Z^	Ref.
	Skins	Seeds	Stems	Marc		
*Flavanols*						
Catechin	-	-	-	1986.00–2124.00	LC-MS/MS	[29]
	0.50–1300.00	158.00–4540.00	60.00–1858.00	87.70–2635.40	HPLC-DAD	[27,30,31,32,34,35,36,38,40,41,43,55,56,57,65,66]
	-	-	-	19.60–89.70	HPLC-DAD-ESi-MSn	[24,33,44,57]
Epicatechin	-	-	-	849.00–1030.00	LC-MS/MS	[27,29,35]
	2.00–850.00	100.00–2700.00	5.00–189.00	57.10–864.70	HPLC-DAD	[30,31,32,34,36,38,41,55,56,57,58,65,66,67]
	-	-	2200.90–3181.50	17.30–112.80	HPLC-DAD-ESi-MSn	[24,33,42,44]
*Hydrolyzable tannins*						
Epicatechin-gallate	-	-	-	91.00–119.00	LC-MS/MS	[29]
	-	1.40–489.00	34.20–130.00	24.40–59.20	HPLC-DAD	[27,30,34,41,65,66]
	-	-	-	10.50–45.60	HPLC-DAD-ESi-MSn	[44]
Epigallocatechin	0.37–0.42	-	-	-	HPLC-DAD-ESi-MSn	[39]
	-	≤129.00	-	-		[30]
Epigallocatechin gallate	0.39–0.49	-	-	-	HPLC-DAD-ESi-MSn	[39]
	-	0.50–156.00	-	-	HPLC-DAD	[30]
Gallocatechin gallate	1.69–2.04	-	-	-	HPLC-DAD-ESi-MSn	[39]
*Condensed tannins*						
Procyanidin B1	-	-	-	467.00–556.00	LC-MS/MS	[29]
	27.00–480.00	29.20–1020.00	133.00–1958.00	10.60–1346.1	HPLC-DAD	[27,30,31,34,36,38,55,56,65,66]
	-	-	228.60–761.20	-	HPLC-DAD-ESi-MSn	[42]
Procyanidin B2	-	-	-	384.00–444.00	LC-MS/MS	[29]
	3.00–650.00	0.80–910.70	11.00–103.00	47.00–244.10	HPLC-DAD	[27,30,31,34,38,41,55,56,57,65,68]
	-	322.00–667.00	-	-	HPLC-DAD-ESi-MSn	[24]
Procyanidin B2-3-*O*-gallate	-	≤738.50	-	-	HPLC-DAD	[65]
Procyanidin B3	0.60–350.00	26.00–315.00	20.00–993.00	9.20–342.00	HPLC-DAD	[27,31,34,36,38,41,55,56,57,65,66]
Procyanidin B4	2.00–300.00	34.00–310.00	30.00–139.00	17.00–515.20	HPLC-DAD	[31,34,37,38,55,65,66]
Procyanidin B5	-	70.00–356.70	-	-	HPLC-DAD	[34]
Procyanidin B7	-	-	-	≤90.00	HPLC-DAD	[36]
Procyanidin B1-gallate	310.00–350.00	660.00–820.00	≤40.00	-	HPLC-DAD	[69]
Procyanidin B2-gallate	23.00–230.00	34.50–505.00	20.00–336.00	135.20–1372.90	HPLC-DAD	[31,36,38,57,66]
Procyanidin B4-gallate	-	9.20–20.80	-	-	HPLC-DAD	[31,36]
Procyanidin C1	10.00–360.00	16.10–600.00	46.00–190.00	17.90–201.10	HPLC-DAD	[27,31,34,36,38,57,66]
Procyanidin C1-gallate	-	15.10–31.90	-	-	HPLC-DAD	[31]
Procyanidin C2	11.00–81.00	152.00–476.00	20.00–115.00	36.50–449.70	HPLC-DAD	[36,38,66]
Procyanidin D1	41.00–320.00	45.00–370.00	10.00–547.00	16.40–852.50	HPLC-DAD	[36,38,66,69]
Procyanidin D2	35.00–79.00	14.00–137.00	37.00–101.00	43.60–266.70	HPLC-DAD	[36,38,66]

^Z^ HPLC-MS/MS, High Performance Liquid Chromatography coupled to Mass Spectrometer; HPLC-DAD, High Performance Liquid Chromatography coupled to Diode Array Detector; HPLC-DAD-ESi-MSn, High Performance Liquid Chromatography coupled to Diode Array Detector, Electro Spray Ionization, and Mass Spectrometer.

**Table 3:** Flavonols present in winery residues.

Compound ^Z^	Concentration (microg·g^−1^ dw)				Analytical Approach ^Z^	Reference
	Skins	Seeds	Stems	Marc		
Astilbin	-	-	-	2.50–7.60	HPLC-DAD-ESi-MSn	[44]
	-	-	≤35.00	-	HPLC-DAD	[40]
Isorhamnetin	-	-	-	12.50–20.50	HPLC-DAD-ESi-MSn	[44]
Isoquercetin	-	-	-	16.00–26.50	HPLC-DAD-ESi-MSn	[44]
I-3-Glc	1.00–23.10	-	-	3.20–63.80	HPLC-DAD	[31,35,36,66,69]
I-3-Fer-Glc	-	-	6.90–9.10		HPLC-DAD-ESi-MSn	[42]
I-3-Gluc	3.50–9.40	-	-	1.90–10.60	HPLC-DAD	[35,36]
Kaempferol	0.20–13.60	-	0.60–15.50	≤2.37	HPLC-DAD	[38,69]
		-	-	9.80–34.20	HPLC-DAD-ESi-MSn	[44]
K-3-Gal	0.10–28.00	-	2.00–15.00	4.00–47.40	HPLC-DAD	[31,35,36]
K-3-Glc	1.90–79.00	-	7.00–26.00	6.00–15.80	HPLC-DAD	[38,58,66]
		-	1.50–8.00	18.40–62.40	HPLC-DAD-ESi-MSn	[36,42]
K-3-Gluc	1.00–19.00	-	1.00–14.00	2.60–13.10	HPLC-DAD	[36,38,66]
K-3-Rut	-	-	1.80–12.10		HPLC-DAD-ESi-MSn	[42]
Laricitrin	-	-	-	0.10–0.30	HPLC-DAD-ESi-MSn	[44]
L-3-Glc	-	-	-	2.90–6.40	HPLC-DAD-ESi-MSn	[44]
	-	-	-	≤3.84	HPLC-DAD	[69]
Myrcetin	-	-	-	2.20–7.20	HPLC-DAD-ESi-MSn	[44]
M-3-Gal	1.90–3.90	-	-	-	HPLC-DAD	[35]
M-3-Glc	2.40–13.80	-	-	≤21.30	HPLC-DAD	[31,35,69]
	-	-	-	3.60–11.40	HPLC-DAD-ESi-MSn	[44]
M-3-Gluc	-	-	-	0.50–1.80	HPLC-DAD-ESi-MSn	[44]
Quercetin	4.00–21.00	1.00–16.00	-	495.00–634.00	LC-MS/MS	[29,38]
	-	-	-	≤15.30	HPLC-DAD	[69]
	-	-	-	93.00–163.60	HPLC-DAD-ESi-MSn	[44]
Q-3-Gal	2.20–22.10	-	3.00–20.00	12.20–63.80	HPLC-DAD	[31,35,36,66]
Q-3-Glc	-	-	-	389.00–704.00	LC-MS/MS	[29]
	0.90–200.00	≤10.00	18.00–170.00	26.00–549.70	HPLC-DAD	[31,35,36,38,40,58,66,69]
	-	958.00–12,744.00	-	-	HPLC-DAD-ESi-MSn	[24]
Q-3-Gluc	-	-	-	243.00–424.00	LC-MS/MS	[1]
	66.80–184.20	0.20–9.00	≤200.00	93.50–522.50	HPLC-DAD	[31,36,38,68,69]
	-	2.60–33.30	42.60–141.90	31.90–81.40	HPLC-DAD-ESi-MSn	[24,42,44]
Q-3-Pen	0.40–12.80	-	-	1.80–4.00	HPLC-DAD	[36,38,66]
Q-3-Rut	-	-	-	27.00–70.00	LC-MS/MS	[29]
	18.00–570.40	25.70–90.50	14.00–21.00	13.00–414.30	HPLC-DAD	[32,36,38,43,66]
	-	0.40–3.30	2.00–9.70	-	HPLC-DAD-ESi-MSn	[24,42]
Syringetin	-	-	-	0.40–0.50	HPLC-DAD-ESi-MSn	[44]
S-3-Glc	-	-	-	4.20–12.00	HPLC-DAD-ESi-MSn	[44]

^Z^ Fer, feruloyl; Gal, galactoside; Glc, glucoside; Gluc, glucoronide; I, isorhamnetin; K, kaempferol; L, larcitrin; M, myrcetin; Pen, pentoside; rha, rhamnoside; Rut, rutinoside; S, syringetin. ^Y^ HPLC-MS/MS, High Performance Liquid Chromatography coupled to Mass Spectrometer; HPLC-DAD, High Performance Liquid Chromatography coupled to Diode Array Detector; HPLC-DAD-ESi-MSn, High Performance Liquid Chromatography coupled to Diode Array Detector, Electro Spray Ionization, and Mass Spectrometer.

**Table 4:** Anthocyanidins present in winery residues.

Compound ^Z^	Concentration (microg·g^−1^ dw)			Analytical Approach ^Y^	Reference
	Skins	Stems	Marc		
D-3-Glc	39.30–142.10	-	-	HPLC-DAD	[35]
C-3-Glc	3.40–83.90	-	-	HPLC-DAD	[35]
Mv-3-Glc	51.70–124.80	-	-	HPLC-DAD	[35]
	-	22.90–80.20	55.80–142.20	HPLC-DAD-ESi-MSn	[43,45]
P-3-Glc	45.80–220.40	-	-	HPLC-DAD	[35]
	-	-	0.80–1.70	HPLC-DAD-ESi-MSn	[45]
Pt-3-Glc	507.60–684.80	-	-	HPLC-DAD	[35]
	-	-	0.10–0.90	HPLC-DAD-ESi-MSn	[45]
D-3-Ac-Glc	2.20–15.50	-	-	HPLC-DAD	[35]
C-3-Ac-Glc	2.20–12.40	-	-	HPLC-DAD	[35]
Mv-3-Ac-Glc	12.20–29.80	-	-	HPLC-DAD	[35]
	-	-	28.40–195.00	HPLC-DAD-ESi-MSn	[45]
P-3-Ac-Glc	9.70–36.20	-	-	HPLC-DAD	[35]
	-	-	0.20–3.30	HPLC-DAD-ESi-MSn	[45]
Pt-3-Ac-Glc	156.10–300.70	-	-	HPLC-DAD	[35]
	-	-	0.03–0.90	HPLC-DAD-ESi-MSn	[45]
C-3-Cou-Glc	2.10–8.90	-	-	HPLC-DAD	[35]
D-3-Cou-Glc	-	-	0.30–43.90	HPLC-DAD-ESi-MSn	[45]
Mv-3-Caff-Glc	-	-	2.60–23.80	HPLC-DAD-ESi-MSn	[45]
Mv-3-Cou-Glc	0.60–3.30	-	-	HPLC-DAD	[35]
	-	-	67.50–238.90	HPLC-DAD-ESi-MSn	[45]
P-3-Cou-Glc	0.70–7.50	-	-	HPLC-DAD	[35]
	-	-	1.60–42.70	HPLC-DAD-ESi-MSn	[45]
Pt-3-Cou-Glc	21.90–33.90	-	-	HPLC-DAD	[35]
	-	-	1.40–72.90	HPLC-DAD-ESi-MSn	[45]

^Z^ Ac, acetyl; C, cyanidin; Cou, coumaroyl; D, delphinidin; Glc, glucoside; Gluc, glucuronide; Mv, malvidin; P, peonidin; Pt, petunidin. ^Y^ HPLC-DAD, High Performance Liquid Chromatography coupled to Diode Array Detector; HPLC-DAD-ESi-MSn, High Performance Liquid Chromatography coupled to Diode Array Detector, Electro Spray Ionization, and Mass Spectrometer.

**Table 5:** Stilbenes present in winery residues.

Compound	Concentration (microg·g^−1^ dw)			Analytical Approach ^Z^	Reference
	Skins	Stems	Marc		
*trans*-Piceid	≤6400.00	74.00–266.00	-	HPLC-DAD	[40,75]
*trans*-Resveratrol	90.00–124,100.00	-	5.80–64.00	HPLC-DAD	[32,37,55]
	-	-	29.00–53.00	HPLC-DAD-ESi-MSn	[43]
Σ-Viniferin	-	167.00–499.00	-	HPLC-DAD	[40]

^Z^ HPLC-DAD, High Performance Liquid Chromatography coupled to Diode Array Detector; HPLC-DAD-ESi-MSn, High Performance Liquid Chromatography coupled to Diode Array Detector, Electro Spray Ionization, and Mass Spectrometer.
